# High-gamma and beta bursts in the left supramarginal gyrus can differentiate verbal memory states and performance

**DOI:** 10.3389/fneur.2025.1627528

**Published:** 2025-07-31

**Authors:** Shennan Aibel Weiss, Nicolás Sawczuk, Daniel Y. Rubinstein, Michael R. Sperling, Katrina Wendel-Mitoraj, Päivi Österman, René Dumay-Roscher, Charles B. Mikell, Sima Mofakham, Kelly Coulehan, Petar M. Djuric, Diego Fernandez Slezak, Juan Esteban Kamienkowski

**Affiliations:** ^1^Department of Neurology, Stony Brook University, Stony Brook, NY, United States; ^2^Department of Computer Science, University of Buenos Aires, Buenos Aires, Argentina; ^3^Department of Neurology and Neuroscience, Thomas Jefferson University, Philadelphia, PA, United States; ^4^Soenia^®^ by Braincare Oy, Tampere, Finland; ^5^Department of Neurosurgery, Stony Brook University, Stony Brook, NY, United States; ^6^Department of Electrical & Computer Engineering, Stony Brook University, Stony Brook, NY, United States

**Keywords:** verbal memory, left supramaginal gyrus, posterior parietal cortex, encoding, recall

## Abstract

**Introduction:**

The left supramarginal gyrus (LSMG) contributes to attentional allocation for memory encoding and may also reflect memory state and performance. Given the roles of high-gamma and beta bursts in cognition and memory, this proof-of-concept study investigated whether these signals within the LSMG could classify memory state and performance.

**Methods:**

Using secondary data from 103 epilepsy patients undergoing presurgical iEEG evaluation, we analyzed 141 delayed verbal free recall experiments. Intracranial EEG (iEEG) data, recorded solely from LSMG electrode contacts, were processed to create two-dimensional (2D) tensors of convolved high-gamma (HG), and beta (15–40 Hz) burst activity. Convolutional neural networks (CNNs) were trained and cross-validated on these 2D tensors to classify memory state (encoding versus recall) and performance (remembered versus forgotten items) within subjects.

**Results:**

The latter CNN, used to label subsequently recalled words based on iEEG recorded during the encoding epoch, performed at or below chance in 79 of the 141 experiments. In all but 3 of these 79 experiments, the iEEG was contaminated or low amplitude. In the other 62 experiments this CNN labeled recalled words with an area under the receiver operating curve (AUROC) score of greater than 0.52. A generalized linear model explained the variance of the AUROC score for labelling recalled words correctly in these 62 experiments (*n* = 62, d.f. = 20, *F* = 1.7, *p* = 1 × 10^−4^). The most significant term in the model was a positive interaction between (1) mean HG burst signal to noise ratio; (2) mean beta burst signal to noise ratio; (3) the number of electrode contacts in the LSMG; and (4) recall probability (*t* = 3.04, *p* = 0.006). We identified 14 experiments that labeled subsequently recalled words during encoding with an AUROC score greater than 0.6. To address over-training, we also trained and then tested the CNN on distinct datasets in four subjects. In most of these experiments CNN performed better than chance. We also found that a CNN utilizing 2D tensors of HG and beta bursts could distinguish encoding from scrambled recall epochs.

**Discussion:**

This work indicates LSMG is a memory hotspot and that HG and beta bursts may serve as temporal memory information packets or signify attention related to memory.

## Introduction

Neurophysiological biomarkers of memory state and performance could prove useful for diagnosing memory disorders and evaluating therapeutics that may slow cognitive decline. Changes in EEG sleep architecture have been found to correlate with tau and *β*-Amyloid as well as memory performance (1–4). On the contrary, memory biomarkers that contemporaneously predict memory performance during task performance are less well understood (5–8). These memory biomarkers could be utilized in clinical trials of drugs that aim to slow or reverse memory disorders, or to improve an individual’s memory performance by triggering closed-loop biofeedback (9) or electrical brain stimulation (6, 10).

The Restoring Active Memory (RAM) project consortium pioneered investigations into neurophysiological biomarkers of verbal memory during task performance. This project utilized intracranial electroencephalography (iEEG) recordings from patients with medically refractory epilepsy as they performed semi-automated verbal memory tasks. Analyses of these recordings, led by investigators at the University of Pennsylvania, utilized logistic regression models (LRMs). The LRMs were trained and cross-validated on binned power spectra derived from all iEEG recording contacts distributed across diverse neuroanatomical structures during word encoding in the verbal free recall task. The area under the receiver operating characteristic curve (AUROC) value served as the metric for assessing the LRM’s ability to correctly classify the word encoding trials that were subsequently recalled. Across subjects, classification performance significantly exceeded chance with a mean AUROC of 0.63 ± 0.07 (5, 6, 11–13).

Recent investigations have identified specific cerebral regions, designated as “memory hotspots,” wherein distinct iEEG spectral patterns serve as reliable biomarkers for successful verbal memory encoding and recall (10, 14, 15) Leveraging data from the RAM project, our prior work (16) demonstrated that iEEG recorded from the left supramaginal gyrus (LSMG) during the encoding of subsequently recalled words showed a significantly higher group level average of high-gamma (HG) power and less theta power as compared to the iEEG recorded during encoding of forgotten words. This neurophysiological distinction is termed the subsequent memory effect (SME), operationally defined as a statistically significant difference (positive or negative) in the average physiological response elicited by recalled versus forgotten items. In the LSMG during memory encoding, SMEs have been observed in functional magnetic resonance imaging (fMRI) blood-oxygen-level-dependent (BOLD) signals and manifest as either positive or negative SME (17). Likewise, during memory retrieval, SMEs are observed in both BOLD signals (18–20) and HG power (21), however in contrast to memory encoding, the SMEs during retrieval are typically positive. The selection of the LSMG as a possible memory hot spot is further supported by its hypothesized involvement in stimulus-driven, bottom-up attention, a cognitive process considered critical for both memory encoding and cued recall (17–20, 22). Furthermore, the LSMG is integrated within the dorsal language network (22), and exhibits functional parallels to the right hemisphere’s ventral attention network (23). This underscores its significance as a key node for mnemonic processing and as a putative mnemonic accumulator. Within this accumulator, neural activity, reflected in high-gamma (80–200 Hz) and beta (16–30 Hz) oscillatory bursts, may correlate with both memory state and behavioral performance, although not necessarily with the formation of individual memory engrams (17–20, 22).

Building upon our previous investigation of SMEs within the LSMG (16), the present study again focused exclusively on iEEG recordings exclusively from the LSMG during the verbal free recall task obtained by the RAM project consortium. Our primary objective was to determine whether verbal memory states (encoding versus retrieval) and memory performance outcomes (recalled versus forgotten) could be differentiated by machine learning analysis of iEEG recorded solely from the LSMG. Our study used convolutional neural networks (CNNs) that function as sophisticated feature detectors, trained on time series data derived from intracranial electroencephalogram (iEEG) recordings from the LSMG. Before being fed into the CNNs, high-gamma and beta oscillatory bursts were first detected. These detected bursts were then represented as a convolved time series, where the power of the bursts was explicitly encoded within the convolution. This approach allowed the CNNs to learn and identify intricate patterns within these pre-processed time series, effectively using the convolved representation as a rich input for their feature detection capabilities. A similar study, that also utilized the RAM dataset, compared the performance of support vector machines (SVMs) trained and tested on iEEG recorded from the LSMG and the left middle temporal gyrus (LMTG) to label recalled words during encoding. This study found that SVM classification results using iEEG recorded from the LSMG were better than chance but significantly inferior to the SVM using iEEG recording from the LMTG (10).

High-gamma bursts are known to track task related multi-unit activity from neuronal populations (24–27), whereas beta oscillations generated by synchronous activity of inhibitory interneurons are linked to information gating and decrease during changes in cognitive control (28, 29). Previous research has shown that relatively more, or less, high-gamma or beta power correlates with successful encoding or recall in the context of deriving SMEs (16, 21). Our hypothesis was that high gamma and beta bursts are features that serve as temporal information packets that can be decoded by CNNs to define both memory state and performance in single word encoding or free recall trials. This hypothesis is supported by studies in primates (29–32) and in humans (8) concluding that the interplay of high-gamma and beta bursts: (1) gate or prevent sensory interference with working memory; (2) represent brief attractor states representing different memory items; and (3) and occur at an increased rate with an increased working memory load. However, these studies mostly focused on local field potential (LFP) or iEEG recordings from the prefrontal cortex (30, 31, 33, 34). Other studies investigating working memory have examined inter-regional communication of high-gamma and beta (35). Several such studies utilized the DARPA RAM dataset and examined the importance of inter-regional communication using high-gamma, beta, theta, and slower bursts quantified in the iEEG (36–40). During word encoding, hippocampus to prefrontal cortex inter-regional information flow was strongest in the delta-theta band, however prefrontal cortex to hippocampus information flow was strongest in the beta band (36). During both word encoding and recall epochs of the verbal free recall task, a comparison of iEEG signals based on fMRI derived intrinsic networks found a bidirectional increase in beta frequencies between the default node network and other intrinsic networks (37). More relevant to our study investigating high-gamma and beta bursts in the LSMG for defining memory state and performance, inter-regional communication the mesial-temporal lobe and ventral posterior parietal cortex in the beta bands was higher during more successful free recall recordings (38). Lastly, higher directed connectivity in the delta-theta frequencies from the left hippocampus to the LSMG was correlated with verbal free recall task performance (40).

**Figure 1:**
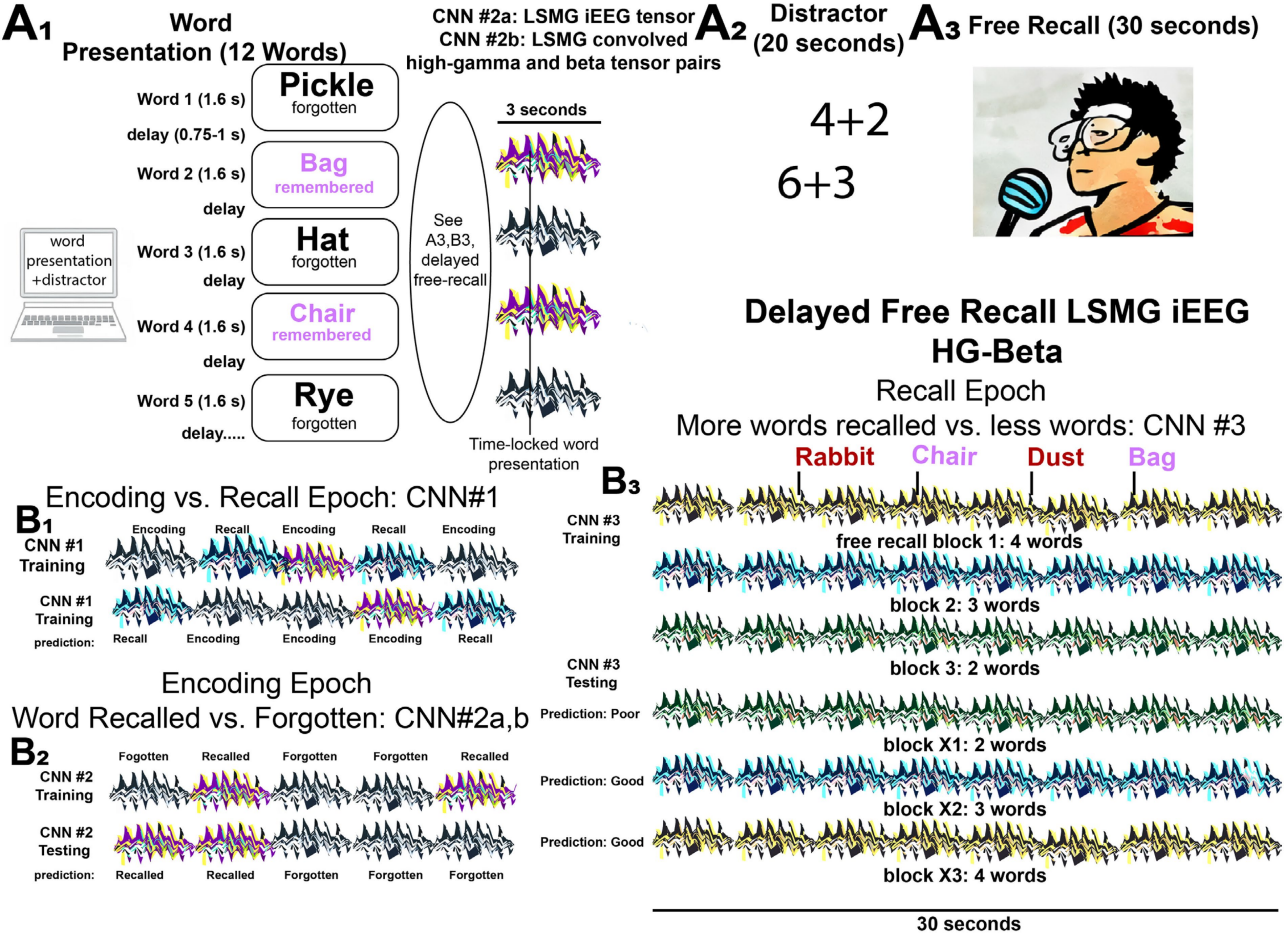
Experimental design for classifying performance in the delayed verbal free recall task using iEEG tensors and convolved high-gamma and beta tensor pairs to train and cross-validate convolutional neural networks (CNNs). **(A)** The patients participated in a verbal delayed free recall task (encoding, distractor, and recall). First, the patient was instructed to remember a list of 12 words that were sequentially presented on a computer screen separated by an inter-word interval **(A1)**. The illustration of LSMG iEEG two-dimensional (2-D) tensors adjacent to the words represent either: (1) 2-D tensor of the broadband iEEG recorded from LSMG contact(s) time locked to word presentation [CNN2a]; or (2) 2-D tensors of convolved high-gamma (HG) and beta time series derived from the LSMG contact(s) that are also time locked with word presentation [CNN2b]. Whether the words, and their respective tensors, correspond with successful or failed word encoding is defined by the delayed free recall epoch **(A3)**, that follows the arithmetic distractor epoch **(A2)**. In the experimental block shown the patient successfully encodes two words “Bag” and “Chair” (magenta). **(B)** Illustrations of the training and cross-validated testing of the 3 CNNs using the 2-D color-coded tensors of convolved high-gamma (HG) and beta burst time series recorded solely from the left supramarginal gyrus (LSMG) intracranial EEG (iEEG) contact(s). Different colors are associated with the different tasks and the task performance (Key: black and white iEEG: encoding—forgotten; purple and yellow: encoding—recalled; black and green: recall—poor; black and blue: recall—good; black and green: recall—good). Note that CNN2a **(B2)** is distinct from CNN1 **(B1)**, CNN2b **(B2)**, and CNN3 **(B3)** because it utilizes the broadband iEEG recorded from the LSMG contact(s) as the 2-D tensor. Additionally, the recall epoch **(B3)** is not time locked to the spoken words and is evaluated solely by the number of words recalled correctly in the 30 s duration. In **(B1)**, the 3 s. word-presentation encoding epochs and compared with scrambled and randomized 3 s epochs from the free recall epoch.

Based upon the hypothesis that features related to beta and high gamma bursts quantified in the LSMG iEEG could decode memory state and performance, we trained several CNNs that utilized iEEG time locked data to specific task epochs (Figure 1, Supplementary Figure 1). The study derived a two-dimensional (2-D) tensor consisting of pairs (one pair for every electrode) of one-dimensional (1-D) tensors: one made of convolved HG bursts; and the other of convolved beta bursts. Thus, the dimensions of the 2-D tensors are two times the number of electrodes and the samples considered for the analysis (see CNN training section in Methods for more detail). These 2-D tensors served as input for training and cross-validating convolutional neural networks (CNNs) designed to classify dichotomized aspects of verbal episodic memory. Specifically, CNNs were developed to distinguish: (1) memory state during encoding versus recall (CNN1, Figure 1B1); (2) encoding performance (successfully encoded versus forgotten words, CNN2, Figure 1B2); and (3) recall performance (trials with higher recall rates compared to other trials, CNN3, Figure 1B3). To further validate CNN2 accuracy, training and testing were also conducted using distinct datasets.

## Methods

### Participants

Patient data were collected as part of a multi-center project named Restoring Active Memory (RAM, Principal Investigator: Michael Kahana) https://memory.psych.upenn.edu/Main_Page#Cognitive_Neuromodulation at the following centers: Thomas Jefferson University Hospital (Philadelphia, PA), University of Texas Southwestern Medical Center (Dallas, TX), Emory University Hospital (Atlanta, GA), Dartmouth-Hitchcock Medical Center (Lebanon, NH), Hospital of the University of Pennsylvania (Philadelphia, PA), and Mayo Clinic (Rochester, MN). The research protocol was approved by the Institutional Review Board at each hospital and informed consent was obtained from each participant. A subset of this data, consisting of 103 subjects and 141 delayed free recall experiments, previously analyzed in (16) and R01 proposal R01MH120161, was utilized by Dr. Weiss and colleagues in accordance with an agreement with Thomas Jefferson University. This data is also available at FR1: https://openneuro.org/datasets/ds004789/versions/3.1.0; catFR1: https://openneuro.org/datasets/ds004809/versions/2.2.0.

### Data preprocessing, anatomical localization, and inclusion criteria

Intracranial electroencephalography (iEEG) was recorded using either subdural grids and strips (10 mm contact spacing) or depth electrodes (3–10 mm contact spacing) at various clinical sites. In each patient, the clinical team determined the placement of the electrodes to best localize epileptogenic regions. The recording systems included DeltaMed & XlTek (Natus), Grass Telefactor, and Nihon-Kohden EEG systems. Sampling rates varied by site, ranging from 500 Hz to 2000 Hz (specifically 500, 512, 1,000, 1,024, or 2,000 Hz). Preprocessing was performed using custom Python software. Individual contact signals were converted to a bipolar montage by calculating the difference between adjacent electrode pairs on each strip, grid, and depth electrode. To remove line noise, the bipolar signal was then notch filtered at 60 Hz using a fourth-order Butterworth filter with a 2 Hz stop-band. Electrode localization involved segmenting hippocampal subfields and medial temporal lobe (MTL) cortices from pre-implant T2-weighted MRIs using multiatlas segmentation (41). Post-implant CT images were manually annotated with electrode coordinates, then coregistered with pre-surgical T1- and T2-weighted scans via Advanced Normalization Tools (42) to align recording sites with anatomical labels. Most MTL depth electrodes, visible on overlaid CT/MRI, were further localized by expert neuroradiologists. Analyzed electrodes are shown in Figure 1 in MNI coordinate space. While automatic segmentation and coregistration, particularly in neurosurgical patients with altered anatomy or displaced tissue from implants, introduce imprecision, the accuracy of our localizations is upheld by the research team’s visual inspection of all alignments and segmentations, and expert neuroradiologist verification of anatomical labels. In this study we included all patients and experiments with at least one subdural or depth contact in the left supramarginal gyrus. We excluded all experiments utilizing intracranial electrical stimulation during the recording.

### Verbal memory task

Each patient participated in a delayed verbal free-recall (FR) task, according to previous experimental settings (5–7, 43–46). An experimental session consisted of encoding, distractor, and recall epochs. The encoding epoch consisted of 12 words, with each word shown on the screen for 1,600 ms, followed by a blank inter-stimulus interval with a duration between 750–1,000 ms. Immediately following the final 12th word in each list, a distractor task consisting of arithmetic problems was performed for 20 s. Subsequently in the recall epoch, participants were then given 30 s to verbally recall as many words as possible from the list in any order (Figure 1A, Supplementary Figure 1). We refer to a single trial of the recall epoch as a single experimental session, to differentiate it from the trials of the encoding epoch. Further details can be found in Ezzyat et al. (5, 6). List words in some experiments were categorically organized (delayed verbal categorical free recall, i.e., catFR). The FR1 and catFR1 experiments were otherwise conducted in an identical manner. Patients completed between 8–50 sessions.

### Convolutional high-gamma (HG) and beta burst time-series analysis

**Figure 2:**
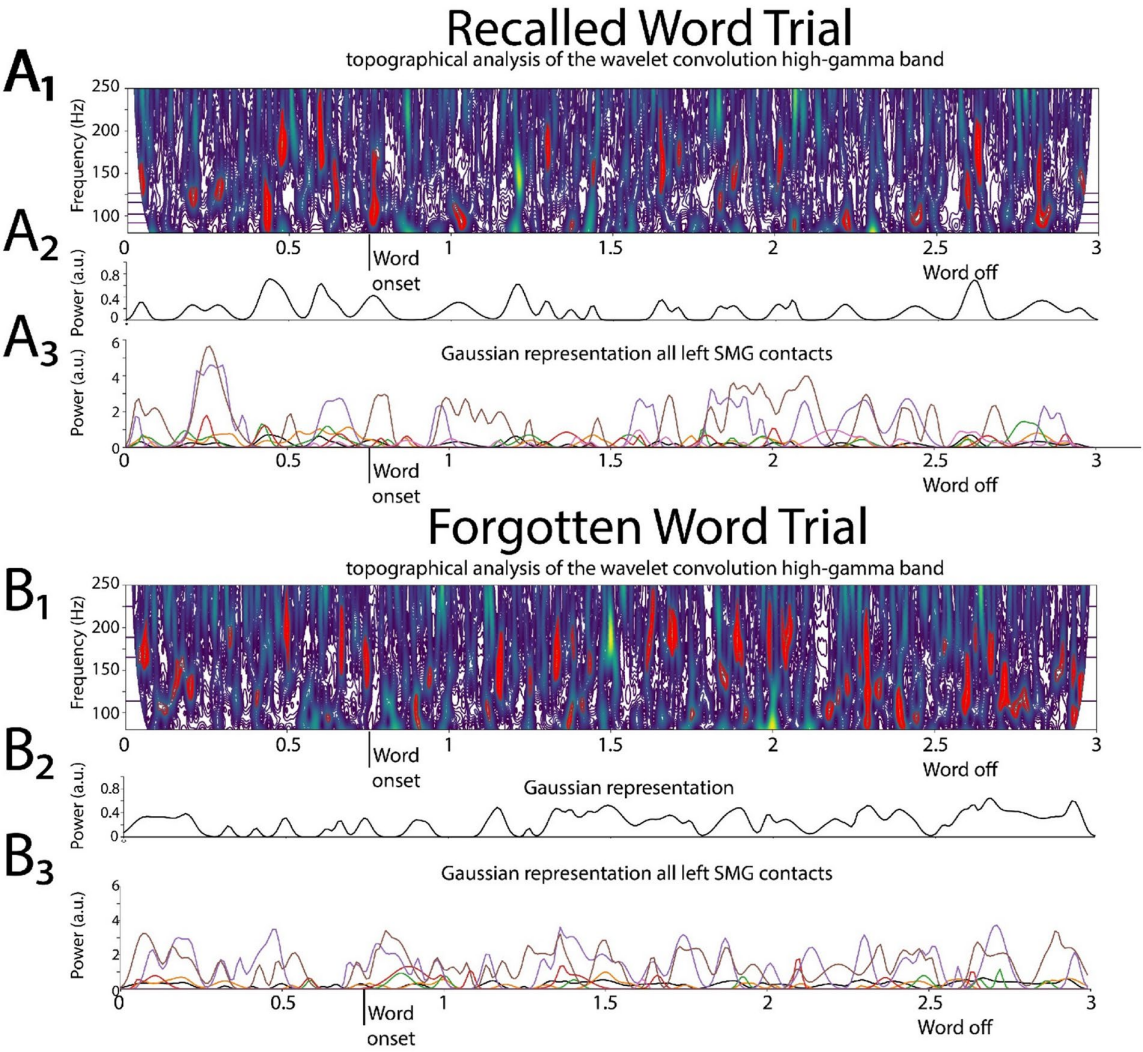
Illustrative example of deriving the two-dimensional (2D) tensor of convolved high-gamma (HG) in the left supramarginal gyrus (LSMG). Shown in **(A1)** and **(B1)** are topographical analyses of the wavelet convolution, in the HG band, during stimulus-locked successful **(A1)** and failed **(B1)** word encoding trials. **(A2)** and **(B2)** are the respective Gaussian convolved time series derived from **(A1)** and **(A2)**, respectively. Shown in **(A3)** and **(B3)** are the Gaussian convolved time series recorded by other contacts in the LSMG used to derive the 2-D tensor of convolved HG, for a successfully encoded **(A)** and forgotten **(B)** word encoding trial.

**Figure 3:**
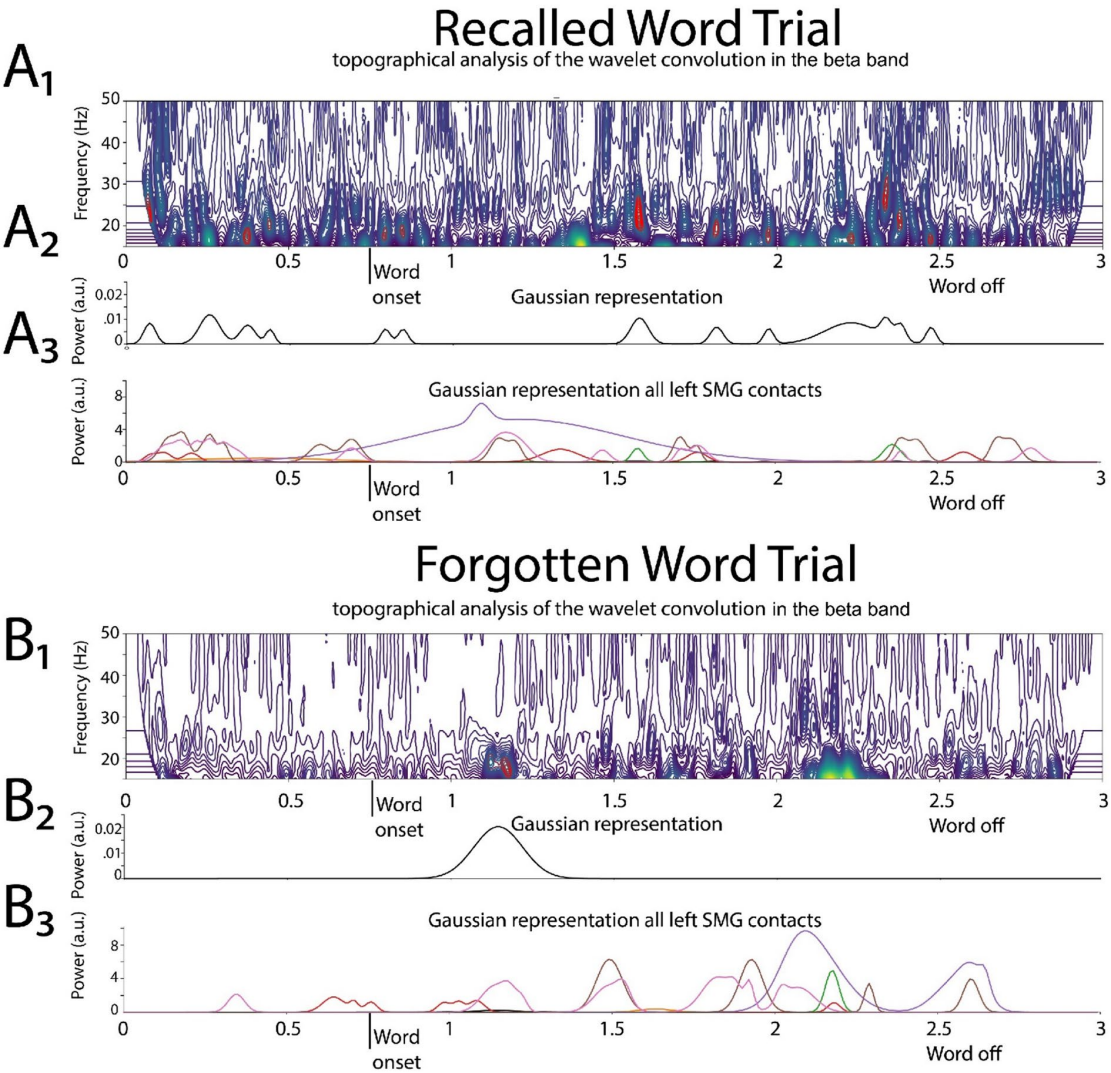
Illustrative example of deriving the two-dimensional tensor of convolved beta in the left supramarginal gyrus (LSMG). Shown in **(A1)** and **(B1)** are topographical analyses of the wavelet convolution, in the beta band, during stimulus-locked successful **(A1)** and failed **(B1)** word encoding trials. **(A2)** and **(B2)** are the respective Gaussian convolved time series derived from **(A1)** and **(A2)**, respectively. Shown in **(A3)** and **(B3)** are the Gaussian convolved time series recorded by other contacts in the LSMG used to derive the 2-D tensor of convolved beta, for a successfully encoded **(A)** and forgotten **(B)** word encoding trial.

All custom code was written in Python™. The signal from each contact was standardized using StandardScaler (scikit-learn). The topographical analysis of the wavelet convolution (47) was adapted to detect the discrete HG and beta oscillatory bursts in the computed wavelet convolutions of the recording from each contact (Figures 2, 3, Supplementary methods). The topographical analysis of the wavelet convolution was utilized twice in frequency ranges overlapping with high-gamma HG [50,250 Hz] and the beta bands [5,50 Hz] to define the onset time, offset time, duration, spectral content, and power of distinct bursts in explicitly in the HG [80–200 Hz] or beta [15–40 Hz] band. Rather than utilizing the raw band-pass filtered iEEG as pairs of 1-D tensors to train CNNs, the oscillatory bursts were represented with Gaussian kernel functions:


$$f(x)=Ae^{\frac{{\left(x-p\right)}^{2}}{2*w^{2}}}$$

where *A* is the amplitude of the Gaussian scaled by the power of each oscillatory burst, *p* the midpoint between onset and offset time and w the time duration of each oscillatory burst.

For a single LSMG iEEG recording from a single contact during a specific epoch of the task, the burst analysis generates both a single HG burst Gaussian (i.e., convolved) time series and a single beta burst Gaussian (i.e., convolved) time series.

### Convolutional neural network (CNN) training and cross-validation

We trained four CNNs: CNN1 distinguished memory state (encoding versus recall), CNN2a distinguished subsequent memory performance (recalled versus unrecalled) of broadband iEEG during encoding, CNN2b distinguished subsequent memory performance based on HG and beta bursts during encoding, and CNN3 distinguished good from poor memory performance based on HG and beta bursts during recall. For all CNNs in this study, excluding CNN2a, the 2-D tensors used for training and cross-validation were constructed by stacking the HG and beta burst time series from each LSMG recording contact. These 2-D tensors had a size equal to twice the number of LSMG contacts.

To train CNN1 to distinguish between encoding and recall (Figure 1B1, Supplementary Figure 1), paired input tensors were created. Each pair comprised two components: (1) a 2D tensor of stacked HG and beta activity from LSMG iEEG during a 3-s, stimulus-locked word encoding trial, and (2) a randomly selected 3-s segment of a similar 2D HG and beta tensor from LSMG iEEG during the subsequent 30-s free recall period. These paired tensors, labeled as “encoding” or “recall,” were randomly concatenated to form the 2D input tensors for training CNN1.

CNN2a and 2b were trained to differentiate between recalled and forgotten words during the stimulus locked word encoding epoch iEEG recordings (Figures 1A2,B2, Supplementary Figure 1). CNN2a was trained using a 2D tensor of the encoding epoch’s word-locked broadband iEEG recordings (the size of the 2D tensor was equal to the number of contacts), which were labeled as encoded or forgotten based on the performance on the free recall block after that session’s distractor (Figure 1A3). CNN2b also classified words as encoded or forgotten but instead utilized the 2-D tensor consisting of convolved HG and convolved beta tensors in the LSMG iEEG during encoding (Figures 1A3,B2).

CNN3 was used to differentiate between good recall and poor recall (Figure 1B3). The LSMG iEEG during the entire 30-s free recall epoch, that followed the distractor, was used to derive a 2D tensor from paired 1-D tensors of convolved HG and convolved beta. Each trial (i.e., session) of the free recall epoch was assigned a binary value of good or poor recall based on whether the number of recalled words exceeded the mode of the number of recalled words across all the experimental sessions. The mode of the number of words recalled varied across the individual patients, with some subjects exhibiting a mode of 0 words recalled.

CNNs were implemented in Keras™ for binary classification. The CNN architecture used for all models consisted of pairs of Conv1D and Batch Normalization layers followed by a Global Average Pooling layer and a fully connected layer as the output layer. The CNN structure utilized for all CNNs was 3 convolutional 1-D layers (with 64, 128 and 64 units per layer), a Kernel size of 3, padding = ‘same’, ReLu activation function and an Adam optimizer. Hyperparameter tuning was performed by testing different structures (consisting of 2 or 3 convolutional layers with 32, 64, 128 or 256 units per layer) and choosing the one that maximized the area under the receiver operating curve (AUROC) across all patients. For the rest of the Conv1D layer parameters, the default values were kept (strides = 1, dilation_rate = 1, groups = 1,use_bias = True, kernel_initializer = “glorot_uniform,” bias_initializer = “zeros,” kernel_regularizer = None, bias_regularizer =None, activity_regularizer = None, kernel_constraint = None, bias_constraint = None). Other hyperparameters were: 100 epochs and a batch size of 128 for CNN1; 150 epochs and a batch size of 128 for analysis for CNN2a,b; and 100 epochs and a batch size of 32 for CNN3. For each patient, for CNN1,2a, and 2b the confusion matrices and related measures were derived using five-fold cross-validation across all the trials. The number of trials for all the CNNs in each fold varied by patient due to variability in the number of experimental sessions completed per patient.

In the case of CNN3 two-fold cross-validation was used because the sessions were relatively low in number (i.e., 12 trials per session). For individual patients the free-recall and categorical free-recall experiments were analyzed separately.

To investigate and account for over-fitting due to the use of cross-validation, for three selected subjects with both FR1 and catFR1 experiments, CNN2b was trained and tested twice: first using the FR1 subset for training and catFR1 for testing, then catFR1 for training and FR1 for testing. The hyperparameters used for this analysis were kept the same as the previous experiments.

The opensource code utilized for the convolutional high-gamma and beta burst time-series analysis and CNN training and cross-validation can be found at https://github.com/nsawczuk/iEEG-experiments.

### LIME for time series

To explain the CNNs predictions for the best performing subject, a modified version of the Local Interpretable Model-agnostic Explanations (LIME) for Time Series method (as described in https://github.com/mdhabibi/LIME-for-Time-Series) was used (48). The method splits one signal into segments and generates several perturbed copies of it (small perturbations are generated on random segments of the signal while leaving the rest unchanged). After predicting the class probabilities for each perturbed copy on a previously trained CNN model, the LIME method fits a linear regression model (with the perturbations and predicted probabilities) and ranks the time segments according to how much they influenced the model output based on the linear regression coefficients. This way the most influential segments (the 20% with the biggest coefficients) for each signal can be identified. To adapt the method to multidimensional data, as it was originally designed for one dimensional time series, the perturbed segments are randomly distributed across all dimensions (each dimension representing a different electrode and high gamma or beta frequency).

### Visual inspection of iEEG

To better understand why most of the patients exhibited CNN2b (AUROC) at chance, or worse, we visually inspected the iEEG recorded from the LSMG in all the subjects during the word encoding epoch. The iEEG recordings were exported from Matlab format to TRC format and visualized in Micromed™ Brain Quick™. The entire duration of each iEEG file was evaluated for excessive 60 Hz line noise, poor grounding resulting in high-frequency noise and muscle artifact, and inter-ictal epileptiform discharges. To estimate the amplitude of HG and beta, the iEEG was band-pass filtered between 80–200 Hz and 15–40 Hz, respectively, using a built-in finite impulse response (FIR) filter in Brain Quick™. The standard cursor, measuring the root-mean-squared (RMS) of the individual filtered signal over a 200 ms window in Brain Quick™, was then used to estimate the amplitude of HG and beta by repeated measures over several minutes of iEEG. Recordings in which the maximum of the RMS HG was consistently less than 5 uV, or of beta less than 10 uV, were labeled as HG and/or beta low-voltage recordings. We presumed these low-voltage recordings may signify contacts in white matter or with abnormal impedance.”

### Generalized linear models (GLM) and high-gamma and beta burst signal to noise analysis

Among the patients in which CNN2b AUROC score exceeded chance (> 0.52) we sought to understand the variables that explained the variance in the CNN2 AUROC score across subjects. We utilized a GLM to fit the CNN2a and CNN2b AUROC using the following pre-defined variables of: (a) electrode type (depth versus subdural electrodes, dummy-coded as 0 and 1 respectively); (b) number of contacts in the LSMG; (c) mean high-gamma burst signal to noise ratio (SNR); (d) mean beta burst SNR; (e) experiment’s correct recall probability; (f) number of word trials. We used the Matlab™ glm.m function with a normal distribution and an identity link, and a fixed intercept. All interaction terms were included. Random effects were not implemented. Using custom Matlab™ code, the HG burst (80–200 Hz) SNR was computed using a 500th-order symmetric FIR filter, while the beta burst (15–40 Hz) SNR was computed using a 100th-order symmetric FIR filter. The root-mean-square (RMS) of each filtered signal served as an estimate of noise. To approximate the signal amplitude, for the derivation of the SNR, we identified peaks in the absolute value of the filtered signals, representing bursts, using second-derivative inflection points above a z-score threshold of 2 (49, 50) and then we measured the amplitude of the 95th percentile of all these bursts. Within each experiment, the SNR for both HG and beta bursts was calculated for each contact and then averaged across all LSMG contacts within patients. These HG and beta burst signal to noise measures were utilized only for the GLM and were not implemented for training and testing the CNNs.

### Statistics

Youden’s J statistic defined as sensitivity + specificity −1, was calculated for each receiver operating curve resulting from cross-validation of the CNNs, or CNNs trained and tested using distinct experiments. Confusion matrix metrics such as sensitivity, specificity, positive predictive value (PPV), negative predictive value (NPV), accuracy, and F1 score were evaluated at the maximum of the Youden J. In addition, the AUROC of CNN2a was compared with the AUROC of CNN2b using the fitlm.m function in Matlab.

## Results

### Labeling remembered words during encoding

The multisite collaborative RAM project examined the word encoding epoch (Figure 1A1) and used binned spectral power of brain activity, across all frequency bands and diverse brain regions, as factors to train a logistic regression classifier to distinguish subsequently recalled words from forgotten words (Figure 1A3). The RAM team found that this method achieved a mean area under the receiver operating curve (AUROC) of 0.63 ± 0.07 (5, 6) using leave-one-out cross-validation. Based on our prior work (16) and the work of others (10), we hypothesized that utilizing recordings solely from the LSMG (Figures 1A1,A3,B2) could achieve comparable performance in classifying remembered words during encoding to the aforementioned RAM studies (5–7). For these experiments we utilized all the RAM experiments with at least one electrode contact in the LSMG.

**Figure 4:**
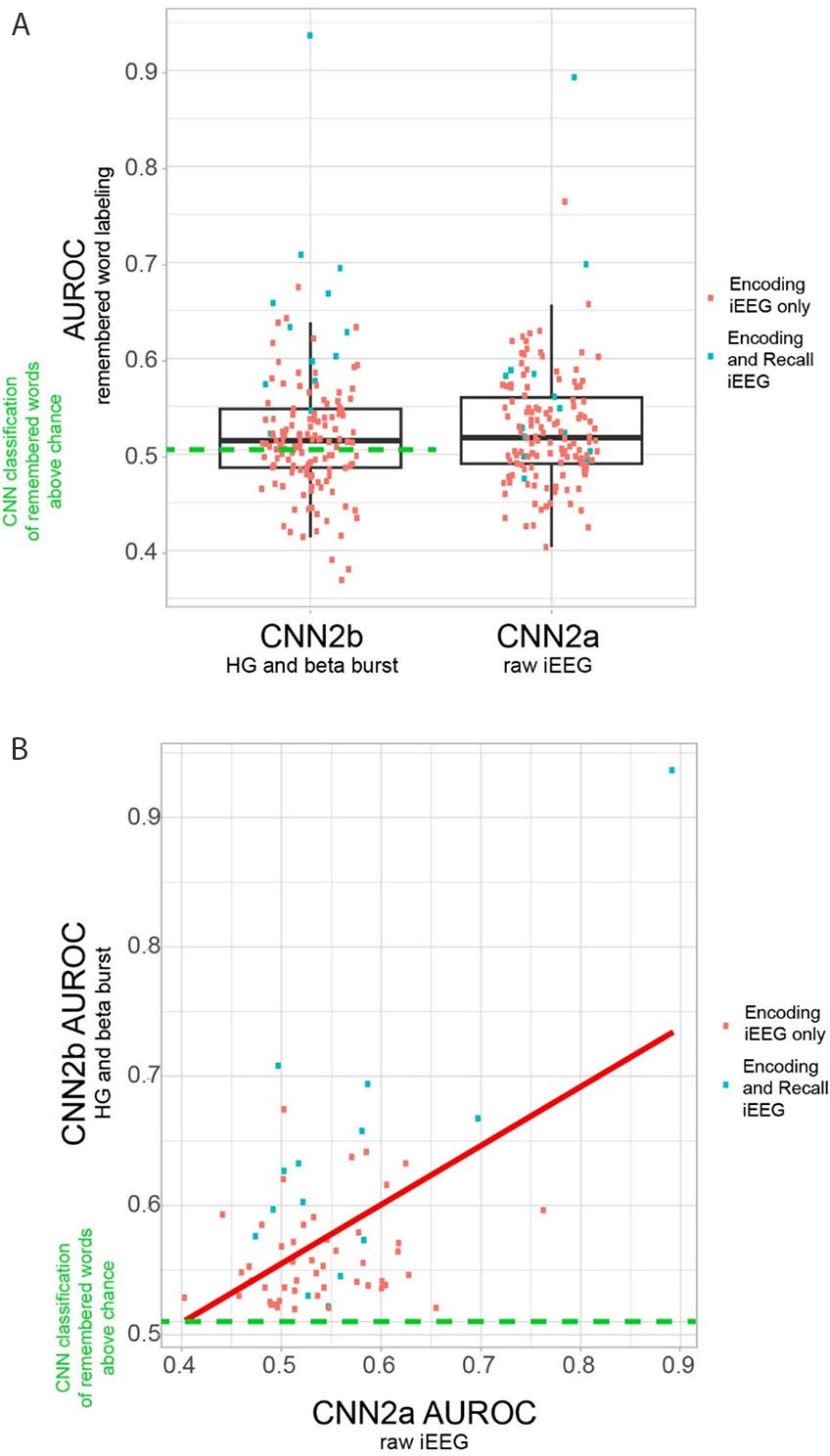
Area under the receiver operating curve (AUROC) for correctly labeling remembered words during encoding by convolutional neural networks (CNN) utilizing either two-dimensional (2-D) tensors of the raw iEEG (CNN2a) or 2-D tensors of convolved bursts of high-gamma and beta (CNN2b) from contact(s) in the left supramarginal gyrus (LSMG). **(A)** Boxplots of the AUROC derived from cross-validation of either CNN2b (left) and CNN2a (right) in *n* = 141 delayed free recall experiments in *N* = 103 subjects (paired *t*-test, *p* > 0.05). Only 62 of the 141 experiments utilizing CNN2b performed better than chance (left, green-dashed-line, AUROC>0.52). The remaining 80 experiments performed at chance or reverse labeled the data (Supplementary Table 1). **(B)** Scatter plot of CNN2b AUROC as a function of CNN2a AUROC (*p* < 1e−5) in the *n* = 62 experiments from *N* = 52 subjects that labeled recalled words during encoding better than chance. In panels A and B, the blue squares indicate experiments that included corresponding recall epoch recordings, and/or distinct encoding training and test sets. Experiments labeled as red squares included only the encoding epoch.

To label subsequently recalled words during the encoding epoch, we trialed two distinct convolutional neural networks (CNNs). CNN2a utilized a 2D tensor of broadband iEEG recorded from the LSMG during the encoding epoch (Figures 1A1,B2, Supplementary Figure 1), and in CNN2b the 2D tensor (Figures 1A1,B2, Supplementary Figure 1) consisted of stacked 1D tensors of convolved high-gamma bursts (Figure 2) and beta bursts (Figure 3) derived from the encoding epoch iEEG. For all the 141 experiments (from 103 subjects see Supplementary Table 1), both CNN2a and CNN2b were trained and then tested using leave-one-out cross-validation. Across all 141 experiments, the mean AUROC score for classifying recalled words during encoding was near chance for both machine learning models (CNN2a AUROC = 0.53; CNN2b AUROC = 0.51, paired *t*-test, *p* > 0.05; Figure 4A).

### Explanatory variables correlating with the variability of within-experiment CNN2 AUROC of remembered word labeling

We found that in 79 of the 141 experiments the within-experiment trained and cross-validated CNN2b labeled recalled words at chance, or reverse labeled words (Figure 4A, Supplementary Tables 1, 2). To understand why, we visually examined the iEEG recordings LSMG in these 79 experiments. We evaluated if these recordings exhibited: (1) minimal amplitude high gamma or beta bursts in 7 experiments; (2) poor grounding resulting in 60 Hz line noise; or excessive high-frequency noise; and/or muscle artifact contamination in 47 experiments; and (3) were contaminated by frequent inter-ictal epileptiform discharges in 9 experiments. Only in 3 of these 79 experiments were these contaminants absent from the iEEG recorded from the LSMG.

We next used generalized linear models (GLMs) to identify explanatory variables for the variance of the CNN2a and CNN2b AUROC across the 62 experiments that labeled recalled words during encoding better than chance (Supplementary Tables 1, 2). The variables used in the GLM included: electrode contact type, number of electrode contacts, mean beta and HG burst signal-to-noise ratios (SNRs), number of word encoding trials, and correct recall probability. For clarity, the value of these mean burst SNRs were incorporated into the GLMs and not utilized for training or testing CNNs. The GLM significantly explained the variance in CNN2b AUROC across subjects (*n* = 62, d.f. = 20, *F* = 1.7, *p* = 1 × 10^–4^), but not CNN2a (*n* = 62, d.f. = 20, F = 1.7, *p* = 0.1). However, AUROCs for CNN2a and CNN2b exhibited a significant positive correlation to each other (*p* < 1 × 10^–5^, Figure 4B). For the GLM explaining the variance of CNN2b AUROC score, the most significant effect was a positive interaction between (1) mean HG burst SNR; (2) mean beta burst SNRs; (3) the number of electrode contacts in the LSMG; and (4) recall probability (Table 1, *t* = 3.04, *p* = 0.006).

**Table 1:** Generalized linear model predicting area under the receiver operator cure for remembered word classification by CNN2b in *n* = 62 experiments and *N* = 52 subjects.

Name	Estimate	SE	tStat	*p*−value
'(Intercept)'	4.07E−01	1.32E−01	3.08E+00	0.006
'e_type'	−5.72E−02	1.06E−01	−5.37E−01	0.597
'e_num'	−8.16E−03	1.06E−02	−7.70E−01	0.45
'hg'	4.48E−02	3.42E−02	1.31E+00	0.206
'beta'	4.33E−03	2.71E−03	1.60E+00	0.126
'recall_p'	9.82E−01	6.99E−01	1.41E+00	0.175
'trials'	6.59E−04	3.26E−04	2.02E+00	0.057
'e_type:e_num'	−4.00E−02	2.89E−02	−1.38E+00	0.182
'e_type:hg'	−1.73E−02	1.09E−02	−1.59E+00	0.128
'e_num:hg'	2.16E−03	2.86E−03	7.56E−01	0.458
'e_type:beta'	4.19E−03	2.30E−03	1.82E+00	0.084
'e_num:beta'	3.24E−04	1.53E−04	2.12E+00	0.047
'hg:beta'	2.27E−04	1.58E−04	1.44E+00	0.166
'e_type:recall_p'	5.39E−01	3.32E−01	1.62E+00	0.12
'e_num:recall_p'	5.54E−02	4.93E−02	1.12E+00	0.274
'hg:recall_p'	−1.89E−01	1.74E−01	−1.08E+00	0.292
'beta:recall_p'	−4.23E−02	1.70E−02	−2.48E+00	0.022
'e_type:trials'	−3.00E−04	1.78E−04	−1.68E+00	0.109
'e_num:trials'	−2.50E−05	1.23E−05	−2.04E+00	0.055
'hg:trials'	−1.81E−04	8.11E−05	−2.23E+00	0.037
'beta:trials'	−1.11E−05	4.68E−06	−2.37E+00	0.028
'recall_p:trials'	−2.32E−03	1.27E−03	−1.83E+00	0.081
'e_type:e_num:hg'	5.75E−03	2.87E−03	2.00E+00	0.059
'e_type:e_num:beta'	2.59E−04	4.45E−04	5.82E−01	0.567
'e_num:hg:beta'	−1.00E−04	3.78E−05	−2.65E+00	0.016
'e_type:e_num:recall_p'	−2.76E−02	3.65E−02	−7.57E−01	0.458
'e_num:hg:recall_p'	−2.10E−02	1.39E−02	−1.51E+00	0.146
'hg:beta:recall_p'	1.10E−04	4.81E−04	2.28E−01	0.822
'e_type:e_num:trials'	7.08E−05	3.96E−05	1.79E+00	0.089
'e_num:hg:trials'	6.17E−06	2.67E−06	2.31E+00	0.031
'hg:recall_p:trials'	6.87E−04	3.41E−04	2.01E+00	0.058
'beta:recall_p:trials'	4.92E−05	2.75E−05	1.79E+00	0.089
'e_type:e_num:hg:beta'	−1.52E−04	1.11E−04	−1.37E+00	0.186
'e_type:e_num:hg:recall_p'	1.33E−02	1.54E−02	8.64E−01	0.398
'e_num:hg:beta:recall_p'	4.57E−04	1.50E−04	3.04E+00	0.006
'e_type:e_num:hg:trials'	−9.13E−06	7.42E−06	−1.23E+00	0.233
'e_num:hg:beta:trials'	1.18E−07	6.96E−08	1.70E+00	0.105
'hg:beta:recall_p:trials'	2.58E−07	5.78E−07	4.47E−01	0.66
'e_type:e_num:hg:beta:recall_p'	1.87E−04	4.76E−04	3.93E−01	0.699
'e_type:e_num:hg:beta:trials'	3.20E−07	1.77E−07	1.81E+00	0.085
'e_num:hg:beta:recall_p:trials'	−6.87E−07	2.97E−07	−2.31E+00	0.032
'e_type:e_num:hg:beta:recall_p:trials'	−1.07E−06	6.53E−07	−1.63E+00	0.118

e_type: depth electrode ‘0’, subdural electrode ‘1’; e_num: # electrode contacts; hg: mean high-gamma signal to noise ration; beta: mean beta signal to noise ratio; recall_p: the correct word recall probability in the experiment; trials: the number of experimental trials. The model had 62 observations, 20 error degrees of freedom, estimated dispersion: 0.00114, F-statistic vs. constant model: 5.2, *p*-value = 1e−4.

### Decoding memory state and performance in selected experiments

**Figure 5:**
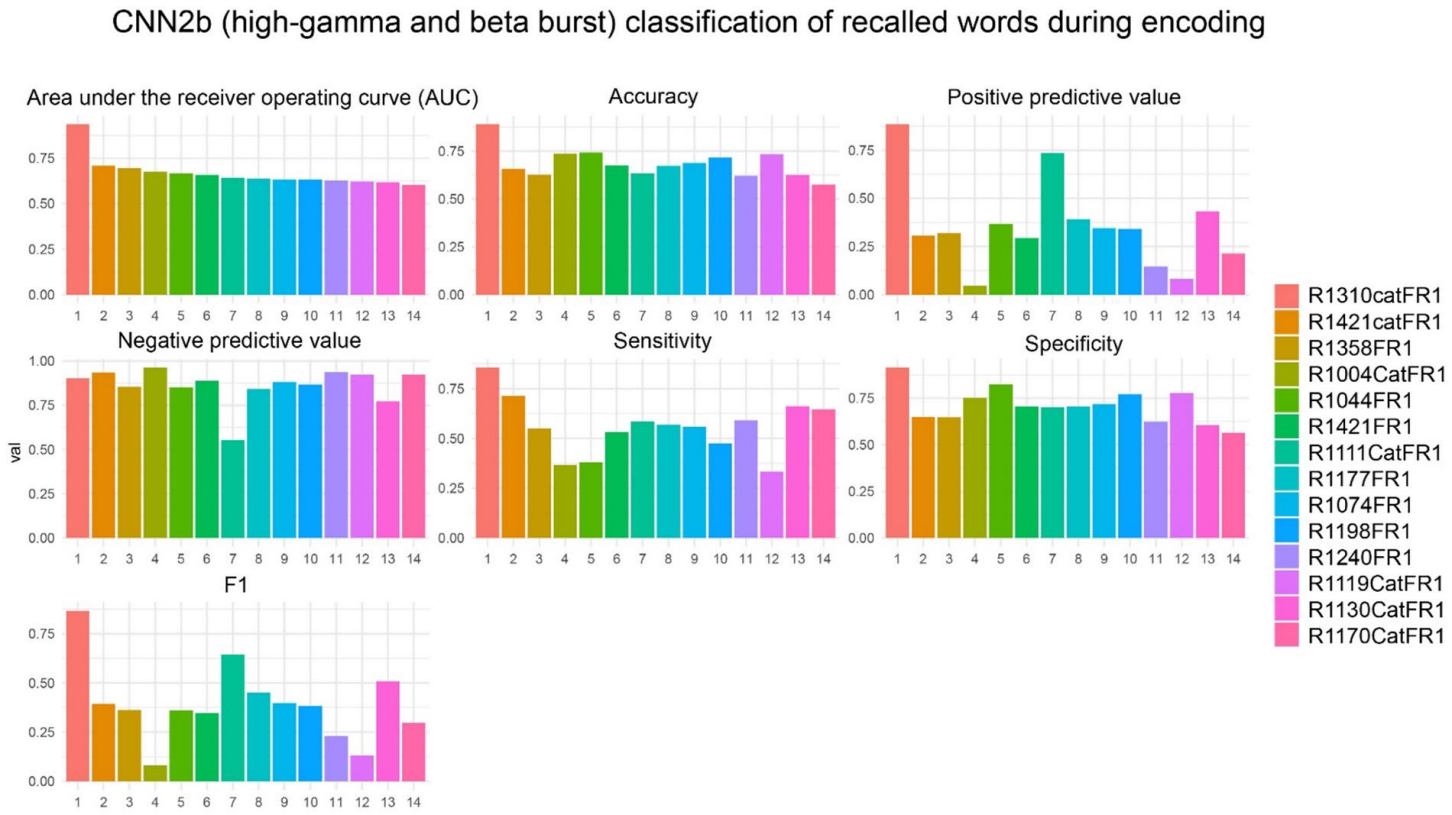
In the best performing subjects, the cross-validation results of convolutional neural network (CNN2b) for correctly labeling remembered words during encoding using a two-dimensional tensor of convolved bursts of high-gamma and beta recorded from contact(s) in the left supramarginal gyrus. The area under the receiver operating curve (AUROC, AUC), confusion matrices values evaluated at the maximum of Youden’s J, and other experimental data in *n* = 14 subjects (see Supplementary Table 1).

**Figure 6:**
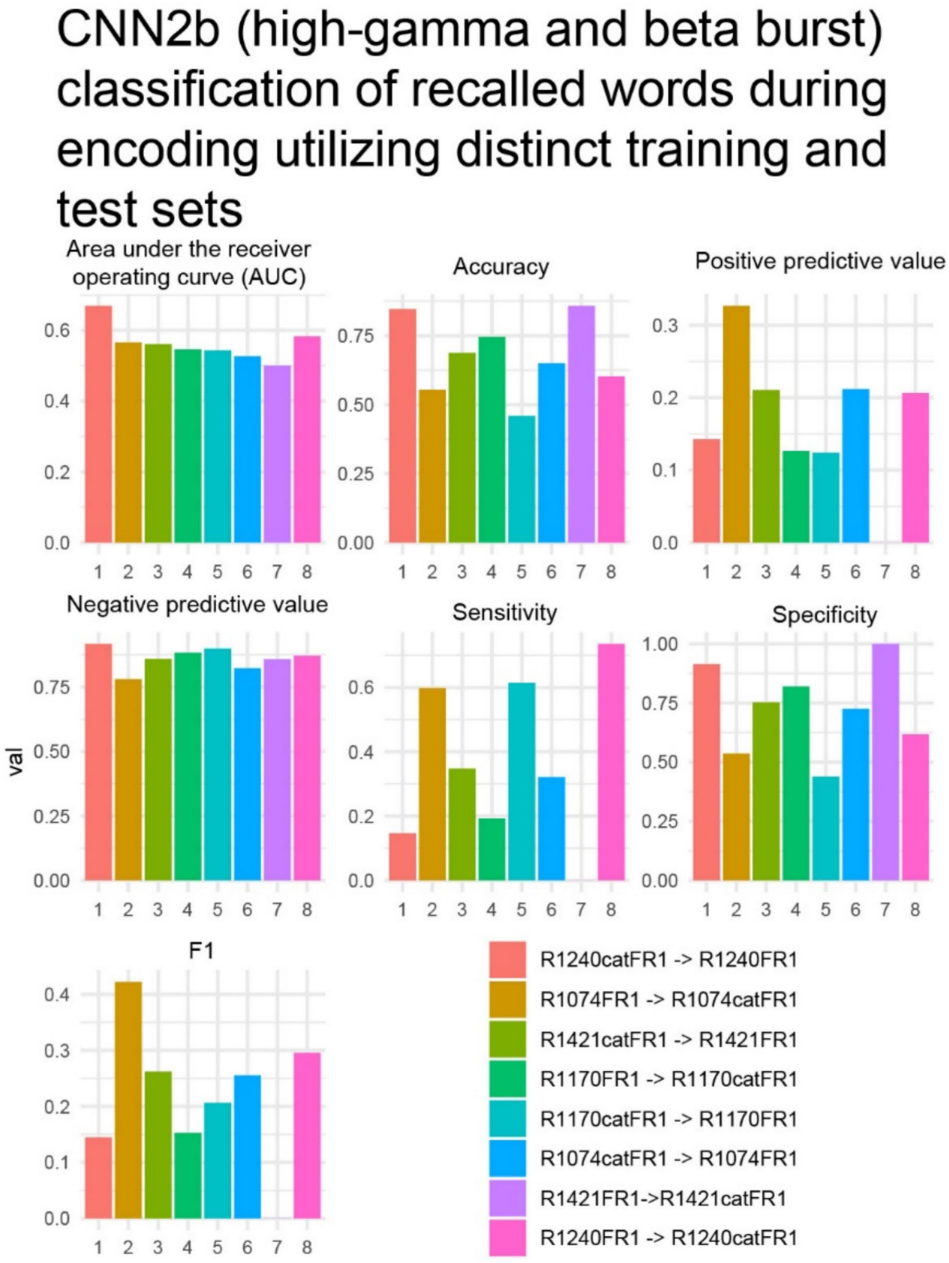
In four subjects with two distinct verbal free recall experiments such as, free-recall [FR] and/or categorical FR [catFR], within patient results of convolutional neural network (CNN2b), trained on one experiment and tested on the other, for correctly labeling remembered words during encoding using a two-dimensional (2-D) tensor of convolved bursts of high-gamma and beta recorded from contact(s) in the left supramarginal gyrus. The area under the receiver operating curve (AUROC, AUC), confusion matrices values evaluated at the maximum of Youden’s J, and other experimental data that are derived from CNN in *n* = 8 experiments and *N* = 4 patients (see Supplementary Table 1). The first experiment in the color code corresponds with the training set, while the second experiment corresponds with the test set. In experiment 7 R1421FR- > R1421catFR the positive predictive value, sensitivity, and F1 value was zero. This implies that the model made no positive predictions or all the positive predictions were incorrect.

To show feasibility supporting our hypothesis that CNNs utilizing paired 2-D tensors of convolved high-gamma and beta bursts exclusively from the LSMG can predict memory state and performance in a dichotomized fashion (Figure 1, Supplementary Figure 1), we selected 14 experiments with a CNN2b AUROC greater than 0.6 (Supplementary Tables 1, 2). Among these 14 patients, we calculated confusion matrix metrics at the maximum of the Youden’s J statistic of the CNN2b receiver operating curve for labeling remembered words during the encoding epoch using five-fold cross-validation of concatenated encoding epoch trials (Figures 1A,B2, Figure 5). To better understand the role of beta bursts in training and testing CNN2b, in three of these experiments, we compared the CNN2b accuracy (Supplementary Figure 2A) and AUROC values (Supplementary Figure 2B) for the CNN2b trained and cross-validated using 2D tensors of just high-gamma bursts as compared to the CNN2b utilizing 2D tensors of both high-gamma and beta bursts. We found that omitting the convolved beta bursts decreased the accuracy of the CNN2b, at the maximum of Youden’s J statistic, for labeling recalled words. However, statistical significance could not be assessed due to the small sample size of only three experiments. To determine whether the AUROC score and accuracy of CNN2b is overestimated due to overtraining in cross-validation, we trained and tested distinct data sets in eight individual experiments from four individual patients (Figures 1A,B2, Figure 6). In this case, the resulting AUROCs were less than the AUROCs derived by five-fold cross-validation. However, CNN2b AUROC score mostly exceeded chance.

The LSMG is part of a frontoparietal network that is important in attention (51), cognition (52), memory (39, 53), and speech comprehension (54). Among the 14 experiments utilizing high-gamma and beta bursts from the LSMG with a CNN2b AUROC greater than 0.6, the study hoped to examine if high-gamma and beta bursts from left pre-frontal and frontal regions can also predict verbal memory state and performance. Unfortunately, the study lacked statistical power. Consequently, the study shifted focus. For nine of the 14 patients, the study compared the CNN2b’s performance when trained and tested on convolved high-gamma and beta bursts from two distinct brain regions: the LSMG iEEG; and left middle temporal gyrus (LMTG) iEEG. The study compared the performance of labeling recalled words during encoding using both AUROC scores and accuracy, determined at the maximum of Youden’s J statistic. The analysis revealed no significant difference in CNN2b AUROC scores between the two recording sites. The AUROC for LSMG iEEG contacts was 0.669 ± 0.034 (mean ± s.e.m.), while for LMTG iEEG contacts, it was 0.611 ± 0.025 (unpaired *t*-test, *t*-stat = 1.372, df = 16, *p* = 0.19; Supplementary Figure 3). Despite similar AUROC values, the CNN2b’s accuracy using LSMG recordings (0.695 ± 0.03) was significantly higher than that using LMTG recordings (0.491 ± 0.051; unpaired *t*-test, *t*-stat = 3.43, d.f. = 16, *p* = 0.0035, Supplementary Figure 3). This suggests that while both regions may offer comparable discriminative power (AUROC), the LSMG recordings provided more precise classifications of successful encoding (Supplementary Figure 3).

**Figure 7:**
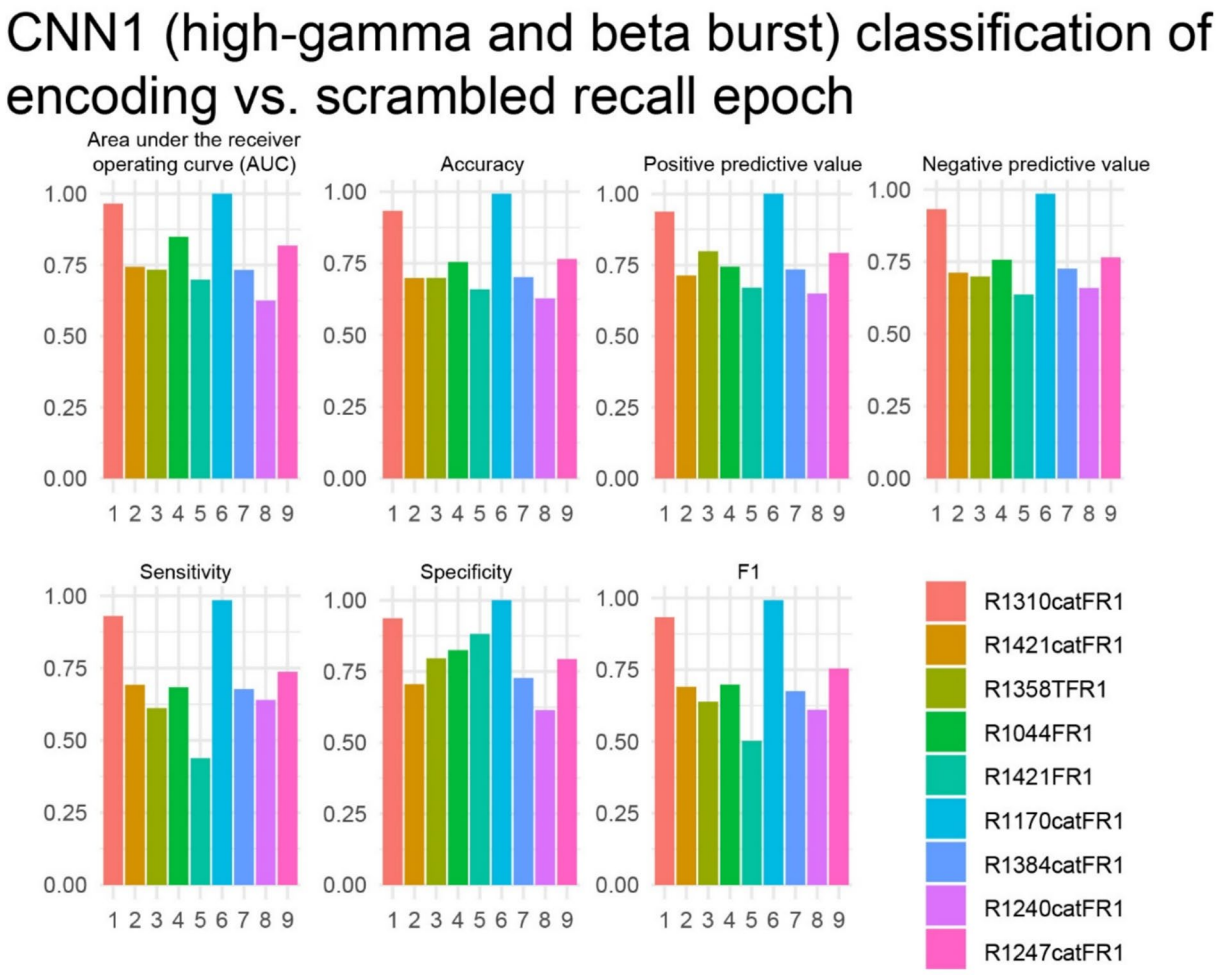
In the best performing subjects, the cross-validation results of convolutional neural network (CNN1) for correctly labeling encoding trials from recall trials using a two-dimensional tensor of convolved bursts of high-gamma and beta recorded from contact(s) in the left supramarginal gyrus. The area under the receiver operating curve (AUROC, AUC), confusion matrices values evaluated at the maximum of Youden’s J, and other experimental data in *n* = 9 subjects (see Supplementary Table 1).

The study next investigated whether iEEG during 3-s word encoding trials could be correctly labeled when scrambled with 3-s iEEG epochs taken from the iEEG recorded during free recall at random by a CNN utilizing paired tensors of bursts of high-gamma and beta in the LSMG iEEG (i.e., CNN1, Figures 1A,B1). Due to the absence of iEEG recordings during the free recall epoch for all experiments (as depicted in Figure 4), subsequent analyses were restricted to a subset of the patient cohort. Specifically, of the 14 patients exhibiting a CNN2b AUROC greater than 0.6, only 9 possessed corresponding iEEG recordings during the free recall period (Supplementary Table 1). In these 9 patients we used five-fold cross-validation to label the encoding epochs and found that the AUROC score was greater than 0.7 in 8 of 9 experiments (Figure 7).

**Figure 8:**
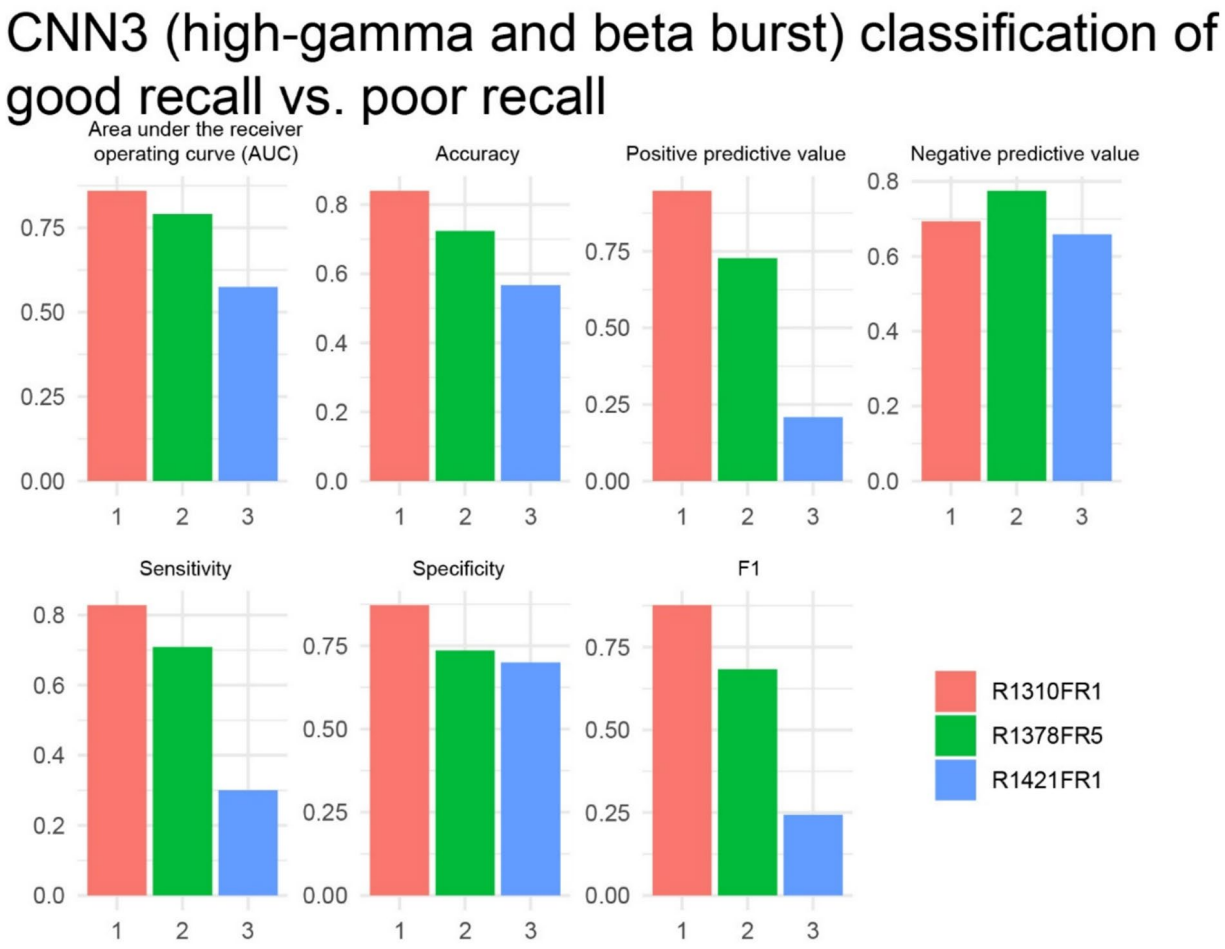
In two of three subjects, the cross-validation results of convolutional neural network (CNN3) for correctly labeling higher performing free recall sessions exceeded chance. The area under the receiver operating curve (AUROC, AUC), confusion matrices values evaluated at the maximum of Youden’s J, and other experimental data in *n* = 3 subjects (see Supplementary Table 1).

Finally, the study analyzed the free-recall epochs within the delayed verbal free recall task. A significant challenge in attempting to label free-recall epochs based on trials with a higher-than-modal number of words recalled, using a machine learning approach, was the limited number of such trials available from individual patients. Specifically, each free-recall session, lasting 30 s, corresponded to an encoding session during which 12 individual word-trials were presented. However, the majority of the 14 subjects participated in fewer than 20 free-recall trials (sessions). We examined three experiments in three subjects with 20 or more free-recall trials. We trained and cross-validated CNN3 (Figures 1A,B3, Supplementary Table 1), which utilizes paired 2-D tensors of convolved high-gamma and beta during the entirety of the 30 s recall epoch, with two-fold cross-validation, to label free-recall epochs in which the subject remembered more words than the mode calculated across all the free-recall trials. We found that in two of the three patients the AUROC score and accuracy at the maximum of Youden’s J provided good classification value (Figure 8). We also asked if the number of words recalled during each free recall session correlated with the total number of words spoken during that free recall session. In the two subjects with a CNN3 AUROC greater than 0.6 we found a strong linear correlation (Supplementary Figure 4, *p* < 0.001) introducing a confound.

**Figure 9:**
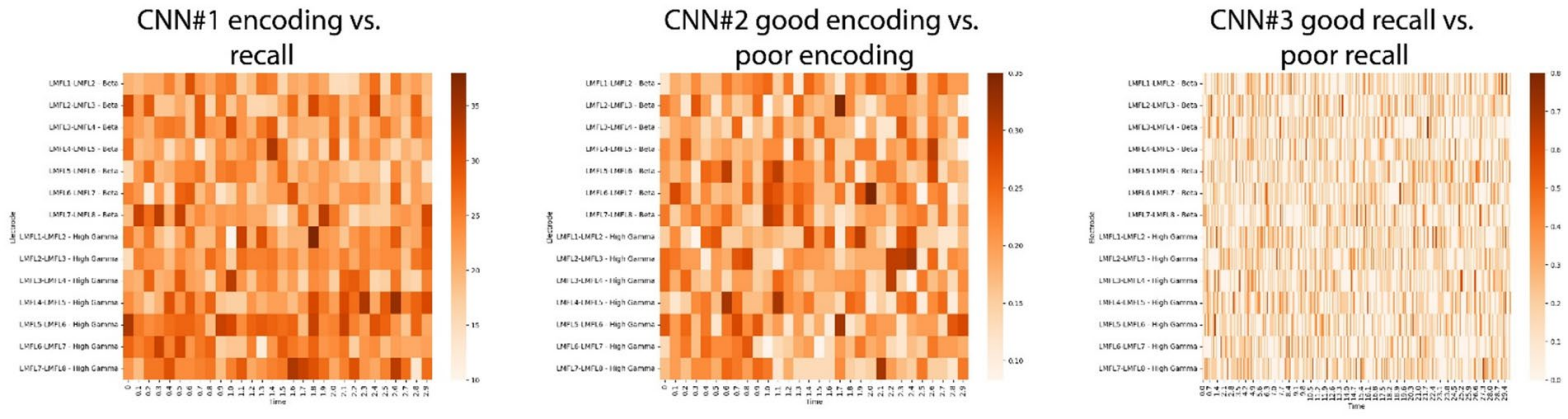
Results of the Local Interpretable Model-agnostic Explanations (LIME) for time series on CNNs for the best performing subject. By generating random perturbations in segments of a signal, the LIME algorithm ranks each segment’s influence on predictions. The method was applied to the CNN1 (**left panel**), CNN2 (**middle panel**) and CNN3 (**right panel**) models of the best performing subject. For every trial in the testing dataset, the most influential segments across all electrodes were identified. The figures show how often each segment in each electrode was considered significant across all trials in the testing dataset.

To facilitate interpretation, a modified version of the LIME for Time Series algorithm was applied to the CNNs trained on a subset of trials from the best-performing subject. This analysis aimed to identify whether specific time segments within particular electrodes significantly contributed to the classification results. The algorithm generates random perturbations within segments of the input signal and quantifies the resulting changes in CNN predictions, thereby identifying influential segments. This process was repeated for all signals in the subject’s testing dataset. Time segments and electrodes were ranked based on their frequency of significance across all trials within this dataset. However, no definitive conclusions could be drawn from these results (Figure 9).

## Discussion

The left supramarginal gyrus (LSMG) is implicated in attentional allocation during memory encoding and may also reflect memory state and performance (17–20, 22). Given the established roles of beta bursts and high-gamma (HG) in working memory (8, 29–35), as well as verbal episodic memory (8, 36–40), this feasibility study investigated whether CNNs could use features related to the time course and amplitude of high-gamma (HG) and beta bursts within the LSMG to classify verbal memory state and performance. Using secondary data from 103 epilepsy patients undergoing pre-surgical iEEG evaluation, we analyzed 141 delayed verbal free recall experiments. Intracranial EEG (iEEG) data, recorded solely from LSMG electrode contacts, were processed to create two-dimensional tensors of convolved high-gamma (80–200 Hz) and beta (15–40 Hz) burst activity. Convolutional neural networks (CNNs) were trained and cross-validated on these tensors to classify encoding versus recall (memory state) and remembered versus forgotten items (memory performance) within subjects. Our exploratory analysis reveals the feasibility of iEEG recordings from the LSMG alone to derive biomarkers of dichotomized verbal episodic memory state and performance during the verbal free recall task.

CNN2b was trained and cross-validated to label recalled words using the time course and amplitude of convolved HG and beta oscillatory bursts extracted from LSMG iEEG recordings during the word encoding epoch of the verbal free recall task. We included all patients with a single intracranial EEG contact localized to the LSMG and excluded experiments that utilized intracranial electrical stimulation. Since experiments with poor iEEG recording quality or contamination with inter-ictal epileptiform spikes (IES) were included, the study found that only 62 of the 141 experimental sessions the CNN2b AUROC score exceeding chance (greater than 0.52), Visual inspection of the iEEG recordings with a CNN2b AUROC score at chance or worse demonstrated poor recordings, contamination, or IES in all but 3 of these experiments. Prior publications utilizing data from the RAM consortium have excluded experiments presumably based on poor recording quality (6, 7, 10).

Among the 141 experiments, 14 experiments exhibited a cross-validated CNN2b AUROC score greater than 0.6 for correctly labeling the subsequently recalled words. This outcome substantiates the feasibility of decoding verbal memory performance, specifically the recall of words during encoding, through analysis of the time course and amplitude of high-gamma (HG) and beta bursts within LSMG intracranial electroencephalography iEEG data. Furthermore, this study mitigates potential confirmation bias through the application of a GLM. The GLM was employed to systematically identify factors explaining the variance in CNN2b AUROC scores across experiments that performed above chance levels. The GLM found that the CNN2b AUROC values were significantly explained by a four-way interaction between HG burst signal-to-noise ratio (SNR), beta burst SNR, the number of electrode contacts within the LSMG, and recall probability (*p* < 0.006). Given that the input tensors for training and cross-validating CNN2b were derived from the delineation and characterization of HG and beta bursts, a diminished SNR in either frequency band could plausibly degrade the CNN2b classification performance. Intriguingly, prior research has indicated that decreased beta activity in the LSMG is associated with cognitive decline (55), suggesting a potential link between beta SNR and mnemonic processing. Furthermore, the significant four-way interaction implies that iEEG signals recorded from distinct subregions within the LSMG provide non-redundant information relevant to classifying memory performance, and consequently, increased spatial sampling within this region enhances the CNN2 AUROC value. Finally, a higher overall recall probability reduces the class imbalance within the dataset (recalled vs. forgotten words), which may also contribute to an improved CNN2b AUROC score. A prior study utilizing the RAM consortium data used synthetic minority over-sampling technique (SMOTE) to address this imbalance in the dataset (10). Should we have utilized SMOTE it would have likely improved CNNs AUROC scores. This same study (10) also raised concerns regarding machine learning over-training in cross-validation. To address this, in a small number of subjects’ experiments, we trained and tested on distinct datasets (i.e., FR1 and catFR1). While these CNN2b AUROC scores were less than the cross-validated CNN2b AUROC score most of the CNN2b trained and tested on the distinct datasets performed better than chance (10).

A prior study compared the performance of a support vector machine (SVM) for classifying memory state and performance across iEEG recordings from diverse neuroanatomical structures. This study found that the left middle temporal gyrus (LMTG) performed best (10). Additional evidence that supports LMTG is the optimal memory hot spot for the verbal free recall task includes prior literature utilizing subsequent memory effect analysis (56–58), and open- (7) and closed-loop (5, 6) electrical stimulation of the LMTG enhancing word encoding performance. Additionally, inter-ictal epileptiform spikes (IES) that are generated during word encoding in the LMTG have the strongest effect size in disrupting recall of the word as compared to IES in other neuroanatomical regions (43). In nine of the 14 patients with an LSMG CNN2b AUROC score greater than 0.6, our study additionally trained and tested a distinct CNN2b using iEEG recordings from the LMTG as a control experiment. Results show that the CNN2b AUROC score did not differ between the CNN2b trained and tested using LSMG iEEG as compared to that using LMTG iEEG. However, the CNN2b accuracy was significantly greater when the CNN2b was trained and tested using iEEG from LSMG as compared to LMTG. The equivalence in the AUROC scores may be due to information carried by beta oscillations in the LMTG (10).

Prior work examining memory encoding and recall has shown the presence of subsequent memory effects (SME) in the LSMG using both fMRI (18–20) and iEEG (16, 21). Delineating a SME relies on averaging responses to repeated stimuli and consequently, many SMEs coincide with stimulus presentation. As a departure from this approach, we investigated whether characterization of distinct HG and beta bursts, that may or may not be, time locked to stimulus presentation, encode or recall individual items in working and episodic memory (31, 32, 46, 59). Our results align with primate studies (29, 31, 32) and studies in humans (8) showing that beta and HG bursts may encode memory related temporal information packets. In support of this notion, human covert and overt speech can also be decoded using bursts of HG in the superior temporal gyrus with a 50% error rate using a 50-word pool size (60). To ask if beta bursts may be redundant with respect to HG bursts, we asked, in three experiments, whether the CNN2b accuracy and AUROC score differed when trained and cross-validated on 2D tensors of convolved high-gamma and beta as compared to high-gamma alone. We found in all three experiments that the accuracy for labeling remembered words decreased with the beta bursts omitted.

It is well established that in cued recall paradigms salient stimuli leads to changes in LSMG activity reflecting the allocation of bottom-up attention that improve recall probability (17–20, 22). Based on these findings, we anticipated that HG and beta bursts in the iEEG in the LSMG during the free recall task would differ between encoding and recall epochs. To train and test this CNN (i.e., CNN1) we derived 2D tensors of stacked 1D tensors of convolved HG bursts and 1D tensors of convolved beta bursts derived from iEEG consisting of intermixed 3 s encoding trials and recall trials. These 3 s recall trials were selected at random from the 30 s recall epoch. We found that CNN1 AUROC score was greater than 0.7 for 8 of 9 of the experiments. Potential confounds include that CNN1 labeled encoding trials by utilizing iEEG signals associated with stimulus preparation and the stimulus itself and identified recall trials based on motor preparation or motor activity during speech. However, our exploratory analysis using LIME argues against this because it showed no specific interval of the encoding vs. recall trial to be most important.

### Limitations

Despite the findings of our study, it remains uncertain whether the HG and beta bursts are causative or simply correlate with respect to verbal memory state and performance. While the LSMG shows significant activity that can be utilized to discriminate memory state and performance, it might be part of a larger network necessary and sufficient for verbal memory. Perhaps the LSMG does not encode or recall the primary verbal memory engram but instead provides necessary attentional input or some other integrative input for this network. Thus, a shortcoming of our study is that we did not examine whether CNN performance may improve if high-gamma and beta burst features were characterized in the LSMG in tandem with that in other neuroanatomical structures. Prior results examining synchronization and desynchronization of different iEEG spectral bands across all neuroanatomical structures during time locked verbal free recall task trials found that successful word encoding is associated with theta band synchronization but gamma and high-gamma band desynchronization (58). An explanation is that high-gamma activity reflects local processing and increases in high-gamma are often temporally dissociated across regions (57). However, characterizing inter-regional transmission of beta bursts as well as bursts of slower oscillations between the LSMG and hippocampus would likely improve the accuracy of decoding memory state and performance (38, 40). Most published research using the RAM consortium data have studied biomarkers of memory state and performance in multiple brain regions together (5–7, 10, 11, 45, 46, 61). In contrast to this important work, our study aimed to utilize the RAM consortium data to demonstrate that the LSMG is a memory hotspot (10, 14, 15). Our implementation and interpretation of CNN1, which distinguishes encoding trials from recall trials, and CNN3, which labels recall sessions in which a greater number of words are recalled, are also limited and have potential flaws. Prior work not only differentiated iEEG recordings of encoding from recall but also included a non-memory related condition (10). Random forest machine learning was utilized to differentiate between these three conditions (i.e., encoding, recall, and non-memory). In our study, CNN1 was dichotomous and differentiated only the encoding from the recall epoch. Moreover, if CNN1 was trained and tested using a cued recall experimental paradigm accurate classification of encoding and recall epochs would be more meaningful. In just two experiments the CNN3 AUROC score was greater than 0.6. However, we found that during the recall epoch the number of words spoken significantly correlated with the number of words recalled. Thus, iEEG contamination from muscle activity, or IEEG activity related to pre-speech or speech may be a confound. However, in cued recall experiments SMEs have been found in the LSMG (21). An investigation of HG and beta bursts in the LSMG during a cued recall paradigm is required to confirm the role of HG and beta bursts in the LSMG in measuring recall performance. Lastly, future investigations of HG and beta bursts in the LSMG as a biomarker of memory state and performance would benefit from comparing several types of machine learning (11).

## Conclusion

Using secondary data from the Restoring Active Memory (RAM) program, we analyzed intracranial EEG recordings from medically refractory epilepsy patients performing a delayed verbal free-recall task. We trained, cross-validated, and tested using distinct experiments, convolutional neural networks (CNNs) using 2D tensors of convolved HG and beta bursts derived exclusively from the iEEG recorded from the LSMG. The CNNs utilized features related to the time course and power of HG and beta bursts to label verbal memory state and performance. Our results demonstrate that, in certain experiments with higher HG and beta SNRs, more electrode contacts placed in the LSMG, and higher recall probably, the AUROC scores for labeling memory state and performance were comparable to the mean AUROC scores reported by RAM researchers using iEEG recordings from diverse neuroanatomical sites synergistically. In accord with prior investigations (10, 38, 40) these findings indicate that the LSMG serves as a verbal memory hotspot. Other brain regions, specifically the left middle temporal gyrus, have also been implicated as memory hotspots as well. However, the LSMG may be unique due to its role in directing attention to memory. More work is needed to explore the different features most important for decoding memory hotspots and how their information content can be best combined. In accord with the established role of HG and beta bursts in working memory, an important and unique conclusion from our study is that characterizing the temporal and spectral features of HG and beta bursts alone accurately labels verbal memory state and performance. With respect to future human memory research, LRMs utilizing non-invasive high density scalp EEG have been shown to label recalled words in the verbal free recall task slightly better than chance (62). Perhaps, high-density non-invasive or minimally invasive sub-scalp EEG recordings proximal to the LSMG could also classify memory state and performance. Epilepsy research has shown that in addition to beta frequency, higher frequency (80–150 Hz) ripples can be characterized in the scalp EEG (63). Non-invasive or minimally invasive recordings could may be used for biofeedback (9) or neurostimulation (6, 64) to enhance memory.

## Data Availability

The datasets presented in this study can be found in online repositories. The names of the repository/repositories and accession number(s) can be found in the article/Supplementary material.
