# Supplementary material for: High-gamma and beta bursts in the left supramarginal gyrus can differentiate verbal memory states and performance
Source: Front Neurol. 2025 Jul 31;16:1627528. doi: 10.3389/fneur.2025.1627528 (PMC12352162; doi:10.3389/fneur.2025.1627528)
Supplement: Supplementary file 1 [file Data_Sheet_1.pdf]

Supplementary Methods

High-gamma (HG) and beta burst detection

The wavelet convolution was applied to the signals using the time_frequency.tfr.cwt function from the MNE Python library using complex Morlet wavelets. The frequency ranges were [5,50 Hz] for beta and [50,250 Hz] for HG (width=7 and gwidth=5). Within each window the iso-contours of power in the wavelet convolution were analyzed as described in (Waldman et al., 2018). The closed contour loop groups with an outermost contour that both surpassed a threshold of power (20% of the maximum power of the plot) and was within the desired frequency range (15-40 Hz for beta and 80-200 Hz for HG) were marked as oscillatory bursts. For each discrete beta and HG burst we defined the oscillatory burst power, frequency, and onset and offset times. For the encoding epoch, oscillatory bursts were detected with a sliding window of 0.2 seconds for HG and 0.6 seconds for beta. The sliding windows had an overlap of 0.1 seconds for HG and 0.3 seconds for beta. For the recall epoch, the topographical analysis of iEEG signals was performed similar to the encoding epoch but in overlapping 3 second segments(with 0.6 seconds of overlap) to generate a continuous HG and beta time series.

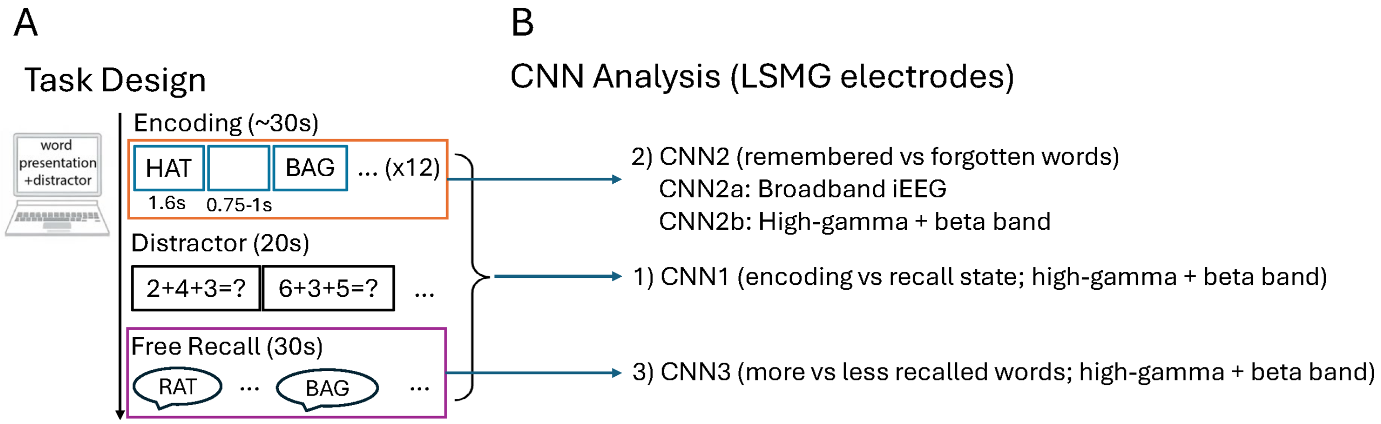

Figure 1. **Experimental design for classifying performance in the delayed verbal free recall task using iEEG tensors and convolved high-gamma and beta tensor pairs to train and cross-validate convolutional neural networks (CNNs)** A) The patients participated in a verbal delayed free recall task (encoding, distractor, and recall). First, the patient was instructed to remember a list of 12 words that were sequentially presented on a computer screen separated by an inter-word interval of 0.75-1s. After a distractor arithmetic task of 20s, the patient was instructed to freely recall as many words as possible for 30s. B1) CNN1 was trained to differentiate encoding epochs from recall epochs based on 2-D tensors (high-gamma, beta band) of left SMG (LSMG) iEEG burst activity. Encoding epochs consisted of 3s word-presentation epochs, and recall training epochs consisted of random 3s epochs from the free recall period. B2) CNNs 2a and 2b were trained on encoding epochs to distinguish subsequently remembered from forgotten words. CNN2a was trained on broadband iEEG burst activity; CNN2b was trained on high-gamma and beta burst activity. B3) CNN3 was trained to distinguish good recall epochs from poor recall epochs, based on high-gamma and beta burst activity, and whether recall number exceeded the mode of the number of recalled words across all sessions for that patient.

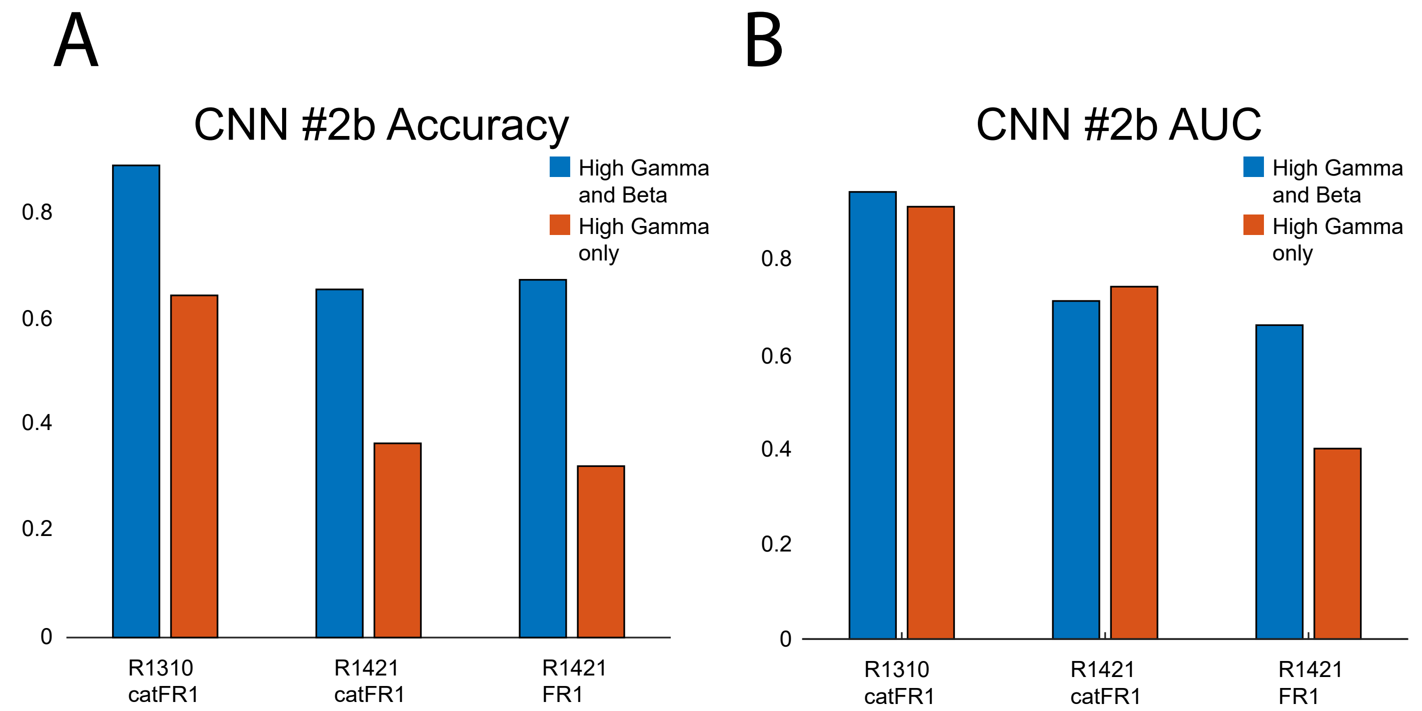

Supplementary Figure 2: A comparison of the accuracy at Youden's J maximum (A) and area under the receiver operating curve (AUROC) score (B) of CNN2b trained and tested with two-dimensional (2D) tensors of both high-gamma and beta bursts (blue) or 2D tensors of high gamma bursts alone (red) derived from the left supramarginal gyrus (LSMG) to label recalled words during the encoding epoch. (A) In three experiments the cross-validated accuracy of CNN2b was higher using the 2D tensors of both high-gamma and beta compared to the 2D tensors of high-gamma alone. (B) The differences in the CNN2b AUROC score across the three patients were inconsistent. Statistical significance could not be assessed due to the small sample size.

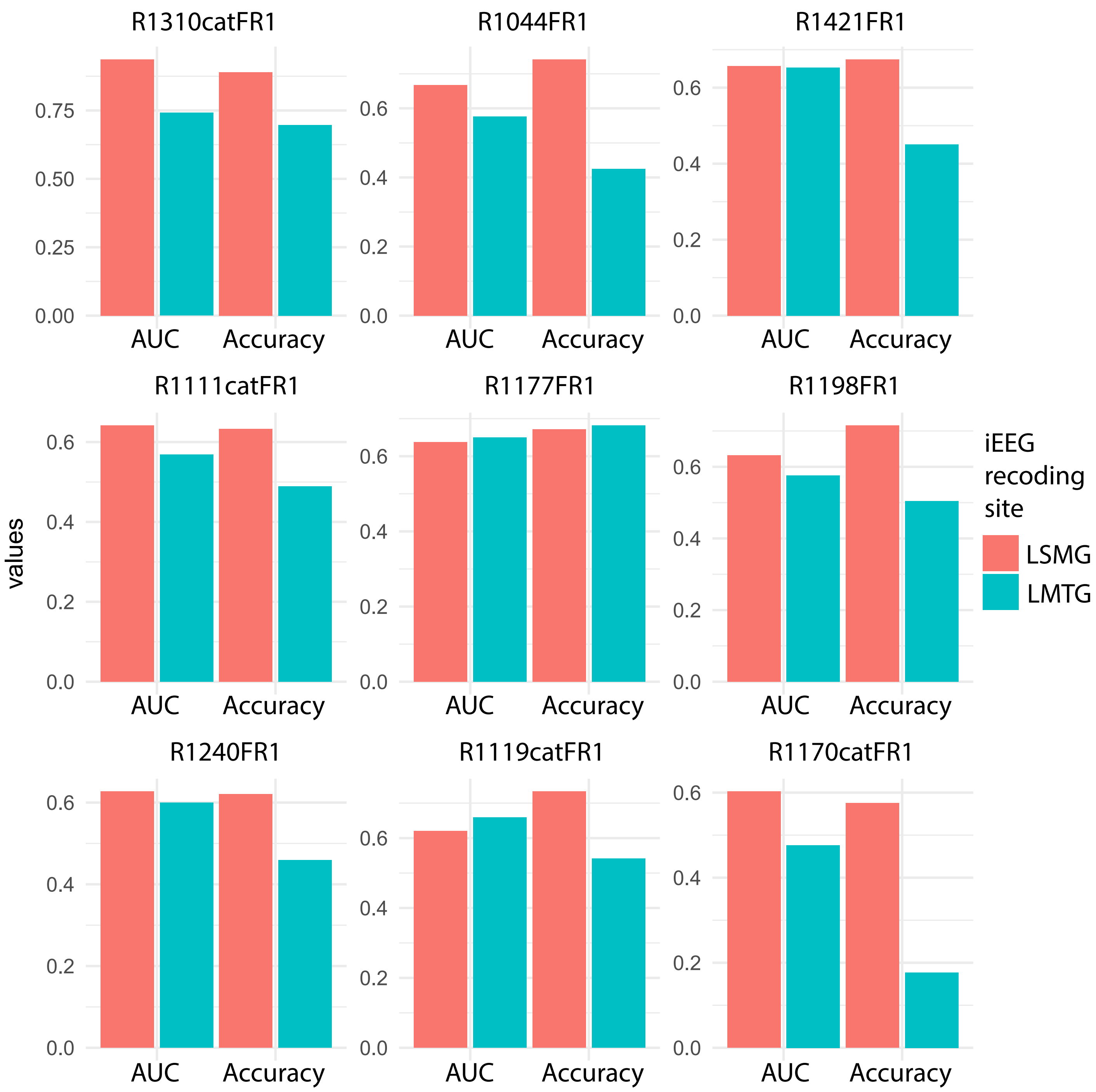

Supplementary Figure 3: A within patient comparison of the area under the operating curve (AUC) score and accuracy, at the maximum of Youden's J, of CNN2b trained and tested with two-dimensional (2D) tensors of both high-gamma and beta bursts derived in iEEG recordings from the left supramarginal gyrus (LSMG, orange) or in iEEG recordings from the left middle temporal gyrus (LMTG, blue). The AUC for LSMG iEEG contacts was 0.669±0.034 (mean ± s.e.m.), while for LMTG iEEG contacts, it was 0.611±0.025 (unpaired t-test, t-stat = 1.372, df=16, p=0.19). Despite similar AUROC values, the CNN2b's accuracy using LSMG recordings (0.695±0.03) was significantly higher than that using LMTG recordings (0.491±0.051; unpaired t-test, t-stat = 3.43, d.f.=16, p=0.0035). A paired t-test was not utilized due to differences of LSMG and LTMG spatial sampling within patients.

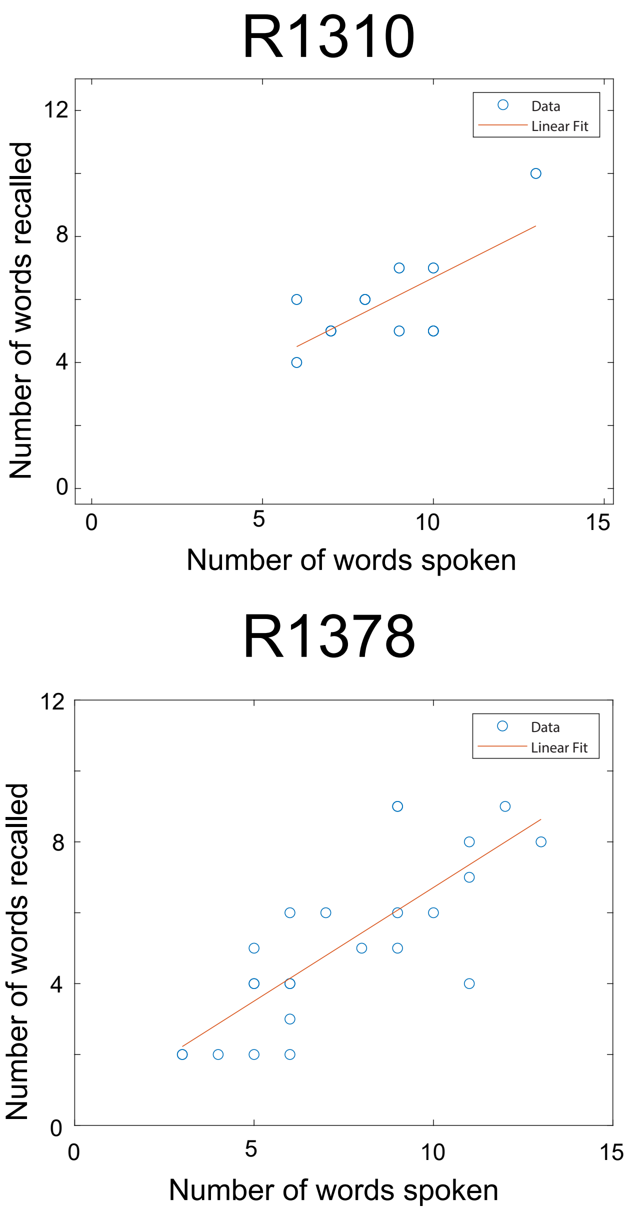

Supplementary Figure 4: In the two patients in which CNN3 labeled categorical free recall sessions with more recalled words with an area under the receiver operating curve of greater than 0.70 the number of words spoken correlated with the number of words recalled across sessions (top, R1310, d.f.=22, adj. R^2=0.477, p=0.0001, bottom R1378, d.f.=22, adj. R^2= 0.6, p<0.0001).

Supplementary Table 1: Complete list of delayed verbal free recall (FR1) and categorical free recall (catFR1) experiments used for secondary analysis of restoring active memory (RAM) data. In each subject tensors of convolved high-gamma and beta bursts were used to train and test convolutional neural networks (CNNs). CNN2b labeled recalled words during encoding and was tested using cross-validation (column 2 and 3) and with distinct training and test sets (column 4). CNN1 was cross-validated to label encoding trials in blocks of randomized (i.e., scrambled) encoding and recall epochs (column 5). CNN3 was cross-validated to label recall sessions in which the subject recalled more words than the mode of all recall trials (column 6). Experiments labeled in green were utilized for CNN analysis that subsequently measured confusion matrices at the maximum of Youden's J of the CNN's area unnder the receiver operating curve (AUROC) greater than 0.6. Experiments labeled in yellow exhibited a cross-validated CNN2b that labeled recalled words better than chance during the encoding epoch. Unlabeled experiment with minus symbol performed at chance or reverse labeled.

experiment name	Cross-validated CNN2b labeling recalled word AUROC greater than chance (AUROC>0.52)	Cross-validated CNN 2b labeling recalled word (AUROC > 0.6)	Distinct training and test set experiments CNN 2b	Cross-validated CNN 1 confusion matrices at maximum of Youden's J	Cross-validated CNN 3 confusion matrices at maximum of Youden's J
'R1310JcatFR1'	x	x		x	x
'R1421McatFR1'	x	x	pair 4b	x	x
'R1358TFR1'	x	x		x	
'R1044JFR1'	x	x		x	
'R1421MFR1'	x	x	pair 4a	x	
'R1170JcatFR1'	x	x	pair 3b	x	
‘R1130McatFR1’	x	x		x	
'R1240TcatFR1'	x	x	pair 1b	x	
‘R1119PcatFR1’	x	x		x	
R1240TFR1'	x		pair 1a		
R1170JFR1	x		pair 3a		
R1074MFR1	x	x	pair 2a		
R1074McatFR1	x		pair 2b		
R1378T_catFR5	n/a "stim"	n/a	n/a	n/a	x "no stim"
'R1004DcatFR1'	x	x			
'R1111McatFR1'	x	x			
'R1177McatFR1'	x	x			
'R1198MFR1'	x	x			
‘R1247PcatFR1’	x				
‘R1384JcatFR1’	x				
'R1086McatFR1'	x				
'R1260DFR1'	x				
'R1003PFR1'	x				
'R1260DcatFR1'	x				
'R1111MFR1'	x				
'R1114CcatFR1'	x				
'R1330DcatFR1'	x				
'R1241JFR1'	x				
'R1221PFR1'	x				
'R1227TcatFR1'	x				
'R1010JFR1'	x				
'R1214MFR1'	x				
'R1346TFR1'	x				

Supplementary Table 1 (continued): Complete list of delayed verbal free recall (FR1) and categorical free recall (catFR1) experiments used for secondary analysis of restoring active memory (RAM) data. In each subject tensors of convolved high-gamma and beta bursts were used to train and test convolutional neural networks (CNNs). CNN2b labeled recalled words during encoding and was tested using cross-validation (column 2 and 3) and with distinct training and test sets (column 4). CNN1 was cross-validated to label encoding trials in blocks of randomized (i.e., scrambled) encoding and recall epochs (column 5). CNN3 was cross-validated to label recall sessions in which the subject recalled more words than the mode of all recall trials (column 6). Experiments labeled in green were utilized for CNN analysis that subsequently measured contingency table matrics at the maximum of Youden's J of the CNN's area unnder the receiver operating curve (AUROC). Experiments labeled in yellow exhibited a cross-validated CNN2b that labeled recalled words better than chance during the encoding epoch. Unlabeled experiment with minus symbol performed at chance or reverse labeled.

experiment name	Cross-validated CNN2b labeling recalled word AUROC greater than chance (AUROC>0.52)	Cross-validated CNN 2b labeling recalled word (AUROC > 0.6)	Distinct training and test set experiments CNN 2b	Cross-validated CNN 1 confusion matrices at maximum of Youden's J	Cross-validated CNN 3 confusion matrices at maximum of Youden's J
'R1226DFR1'	x				
'R1159PFR1'	x				
'R1203TFR1'	x				
'R1107JcatFR1'	x				
'R1059JFR1'	x				
'R1323TFR1'	x				
'R1114CFR1'	x				
'R1378TFR1'	x				
'R1291MFR1'	x				
'R1195EFR1'	x				
'R1067PFR1'	x				
'R1286JFR1'	x				
'R1094TcatFR1'	x				
'R1338TcatFR1'	x				
'R1390McatFR1'	x				
'R1232NFR1'	x				
'R1094TFR1'	x				
'R1134TFR1'	x				
'R1108JcatFR1'	x				
'R1217TcatFR1'	x				
'R1136NFR1'	x				
'R1217TFR1'	x				
'R1070TFR1'	x				
'R1052EFR1'	x				
'R1102PFR1'	x				
'R1102PcatFR1'	x				
'R1158TcatFR1'	x				
'R1330DcatFR1'	x				
'R1174TFR1'	x				
'R1108JcatFR1'	x				
R1153TTFR1	-				
R1274TcatFR1	-				
R1154DFR1	-				
R1374TcatFR1	-				

Supplementary Table 1 (continued): Complete list of delayed verbal free recall (FR1) and categorical free recall (catFR1) experiments used for secondary analysis of restoring active memory (RAM) data. In each subject tensors of convolved high-gamma and beta bursts were used to train and test convolutional neural networks (CNNs). CNN2b labeled recalled words during encoding and was tested using cross-validation (column 2 and 3) and with distinct training and test sets (column 4). CNN1 was cross-validated to label encoding trials in blocks of randomized (i.e., scrambled) encoding and recall epochs (column 5). CNN3 was cross-validated to label recall sessions in which the subject recalled more words than the mode of all recall trials (column 6). Experiments labeled in green were utilized for CNN analysis that subsequently measured contingency table matrics at the maximum of Youden's J of the CNN's area unnder the receiver operating curve (AUROC). Experiments labeled in yellow exhibited a cross-validated CNN2b that labeled recalled words better than chance during the encoding epoch. Unlabeled experiment with minus symbol performed at chance or reverse labeled.

experiment name	Cross-validated CNN2b labeling recalled word AUROC greater than chance (AUROC>0.52)	Cross-validated CNN 2b labeling recalled word (AUROC > 0.6)	Distinct training and test set experiments CNN 2b	Cross-validated CNN 1 confusion matrices at maximum of Youden's J	Cross-validated CNN 3 confusion matrices at maximum of Youden's J
R1190PcatFR1	-				
R1021DcatFR1	-				
R1147PcatFR1	-				
R1084TFR1	-				
R1054JFR1	-				
R1124JFR1	-				
R1135EcatFR1	-				
R1216EFR1	-				
R1022JFR1	-				
R1069MFR1	-				
R1317DFR1	-				
R1125TFR1	-				
R1207JcatFR1	-				
R1390McatFR1	-				
R1378TcatFR1	-				
R1317DcatFR1	-				
R1138TFR1	-				
R1379EcatFR1	-				
R1372CcatFR1	-				
R1067PcatFR1	-				
R1130MFR1	-				
R1297TFR1	-				
R1161EFR1	-				
R1167MFR1	-				
R1120EFR1	-				
R1221PcatFR1	-				
R1348JcatFR1	-				
R1101TFR1	-				
R1059JFR1	-				
R1187PFR1	-				
R1374TFR1	-				
R1286JFR1	-				
R1066PcatFR1	-				
R1273DcatFR1	-				

Supplementary Table 1 (continued): Complete list of delayed verbal free recall (FR1) and categorical free recall (catFR1) experiments used for secondary analysis of restoring active memory (RAM) data. In each subject tensors of convolved high-gamma and beta bursts were used to train and test convolutional neural networks (CNNs). CNN2b labeled recalled words during encoding and was tested using cross-validation (column 2 and 3) and with distinct training and test sets (column 4). CNN1 was cross-validated to label encoding trials in blocks of randomized (i.e., scrambled) encoding and recall epochs (column 5). CNN3 was cross-validated to label recall sessions in which the subject recalled more words than the mode of all recall trials (column 6). Experiments labeled in green were utilized for CNN analysis that subsequently measured contingency table matrics at the maximum of Youden's J of the CNN's area unnder the receiver operating curve (AUROC). Experiments labeled in yellow exhibited a cross-validated CNN2b that labeled recalled words better than chance during the encoding epoch. Unlabeled experiment with minus symbol performed at chance or reverse labeled.

experiment name	Cross-validated CNN2b labeling recalled word AUROC greater than chance (AUROC>0.52)	Cross-validated CNN 2b labeling recalled word (AUROC > 0.6)	Distinct training and test set experiments CNN 2b	Cross-validated CNN 1 confusion matrices at maximum of Youden's J	Cross-validated CNN 3 confusion matrices at maximum of Youden's J
R1390MFR1	-				
R1349TFR1	-				
R1229MFR1	-				
R1204TcatFR1	-				
R1277JcatFR1	-				
R1193TFR1	-				
R1026DFR1	-				
R1036MFR1	-				
R1291McatFR1	-				
R1066PFR1	-				
R1167McatFR1	-				
R1069McatFR1	-				
R1080EFR1	-				
R1379EFR1	-				
R1196NFR1	-				
R1118NFR1	-				
R1204TFR1	-				
R1045EcatFR1	-				
R1355TFR1	-				
R1290McatFR1	-				
R1158TcatFR1	-				
R1138TcatFR1	-				
R1286JFR1	-				
R1066PFR1	-				
R1163TFR1	-				
R1030JFR1	-				
R1398JFR1	-				
R1229McatFR1	-				
R1187PcatFR1	-				
R1049JFR1	-				
R1207JFR1	-				
R1398JcatFR1	-				
R1274TFR1	-				
R1015JFR1	-				

Supplementary Table 1 (continued): Complete list of delayed verbal free recall (FR1) and categorical free recall (catFR1) experiments used for secondary analysis of restoring active memory (RAM) data. In each subject tensors of convolved high-gamma and beta bursts were used to train and test convolutional neural networks (CNNs). CNN2b labeled recalled words during encoding and was tested using cross-validation (column 2 and 3) and with distinct training and test sets (column 4). CNN1 was cross-validated to label encoding trials in blocks of randomized (i.e., scrambled) encoding and recall epochs (column 5). CNN3 was cross-validated to label recall sessions in which the subject recalled more words than the mode of all recall trials (column 6). Experiments labeled in green were utilized for CNN analysis that subsequently measured contingency table matrics at the maximum of Youden's J of the CNN's area unnder the receiver operating curve (AUROC). Experiments labeled in yellow exhibited a cross-validated CNN2b that labeled recalled words better than chance during the encoding epoch. Unlabeled experiment with minus symbol performed at chance or reverse labeled.

experiment name	Cross-validated CNN2b labeling recalled word AUROC greater than chance (AUROC>0.52)	Cross-validated CNN 2b labeling recalled word (AUROC > 0.6)	Distinct training and test set experiments CNN 2b	Cross-validated CNN 1 confusion matrices at maximum of Youden's J	Cross-validated CNN 3 confusion matrices at maximum of Youden's J
R1086MFR1	-				
R1026DcatFR1	-				
R1373TFR1	-				
R1015JcatFR1	-				
R1164EFR1	-				
R1100DFR1	-				
R1324McatFR1	-				

Supplementary Table 2: Results of visual inspection of the iEEG of experiments exhibiting a CNN2b area under the receiver operating curve of labeling remembered words during encoding at chance or with reverse labeling. The files were inspected for lower amplitude high-gamma and beta bursts, line noise, and high frequency noise resulting from poor grounding or muscle artifact. iEEG files were also inspected for frequent epileptiform discharges. Left supramarginal gyrus recordings iEEG recordings that were used to train and cross-validate convolutional neural networks that performed better than chance were also inspected and did not exhibit these defficiencies.

Subject	# subdural electrodes	# depth electrodes	low power HG and beta bursts	line noise, poor grounding, or muscle artifact	epileptiform discharges
R1153T	5	0			
R1274T	4	0			x
R1154D	0	3	x		
R1374T	2	0			x
R1190P	0	4		x	
R1021D	0	1	x		
R1147P	0	11		x	
R1084T	3	27		x	
R1054J	4	0	x		
R1124J	6	0	x		
R1135E	12	3		x	
R1216E	0	2		x	
R1022J	10	0			x
R1069M	0	21		x	
R1317D	0	13		x	
R1125T	6	0		x	
R1207J	2	0		x	
R1378T	9	0		x	
R1317D	0	13		x	
R1138T	7	0		x	
R1379E	7	0		x	
R1372C	5	0		x	
R1067P	0	7		x	
R1130M	0	11		x	
R1297T	2	0			
R1161E	14	0		x	
R1167M	0	4		x	
R1120E	0	3		x	
R1348J	5	0		x	
R1101T	8	0			x
R1059J	0	2			
R1187P	0	13		x	
R1374T	2	0			x
R1286J	4	0		x	

Supplementary Table 2 (continued): Results of visual inspection of the iEEG of experiments exhibiting a CNN2b area under the receiver operating curve of labeling remembered words during encoding at chance or with reverse labeling. The files were inspected for lower amplitude high-gamma and beta bursts, line noise, and high frequency noise resulting from poor grounding or muscle artifact. iEEG files were also inspected for frequent epileptiform discharges. Left supramarginal gyrus recordings iEEG recordings that were used to train and cross-validate convolutional neural networks that performed better than chance were also inspected and did not exhibit these defficiencies.

Subject	# subdural electrodes	# depth electrodes	low power HG and beta bursts	line noise, poor grounding, or muscle artifact	epileptiform discharges
R1273D	0	3		x	
R1390M	0	1		x	
R1349T	8	0			x
R1229M	5	0		x	
R1204T	5	0			
R1277J	2	0		x	
R1193T	4	0		x	
R1026D	0	5		x	
R1036M	0	11		x	
R1291M	0	15		x	
R1167M	0	4		x	
R1069M	0	21		x	
R1080E	0	3		x	
R1379E	7	0		x	
R1196N	0	7		x	x
R1118N	0	4			
R1204T	5	0		x	
R1045E	0	1		x	
R1355T	2	0		x	
R1290M	8	0			x
R1158T	6	0			
R1138T	9	0		x	
R1286J	4	0		x	
R1163T	13	0		x	
R1030J	6	0	x		
R1398J	2	0		x	
R1229M	5	0		x	
R1187P	0	13		x	
R1049J	13	0		x	
R1207J	2	0		x	
R1398J	2	0		x	
R1274T	4	0			x
R1015J	4	0	x		

Supplementary Table 2 (continued): Results of visual inspection of the iEEG of experiments exhibiting a CNN2b area under the receiver operating curve of labeling remembered words during encoding at chance or with reverse labeling. The files were inspected for lower amplitude high-gamma and beta bursts, line noise, and high frequency noise resulting from poor grounding or muscle artifact. iEEG files were also inspected for frequent epileptiform discharges. Left supramarginal gyrus recordings iEEG recordings that were used to train and cross-validate convolutional neural networks that performed better than chance were also inspected and did not exhibit these defficiencies.

Subject	# subdural electrodes	# depth electrodes	low power HG and beta bursts	line noise, poor grounding, or muscle artifact	epileptiform discharges
R1163T	13	0		x	
R1030J	6	0	x		
R1398J	2	0		x	
R1229M	5	0		x	
R1187P	0	13		x	
R1049J	13	0		x	
R1207J	2	0		x	
R1398J	2	0		x	
R1274T	4	0			x
R1015J	4	0	x		
R1026D	0	5		x	
R1373T	17	0		x	
R1015J	4	0	x		
R1164E	0	3		x	
R1100D	0	1	x		
R1324M	6	0		x	
